# Care practices and neonatal survival in 52 neonatal intensive care units in Telangana and Andhra Pradesh, India: A cross-sectional study

**DOI:** 10.1371/journal.pmed.1002860

**Published:** 2019-07-23

**Authors:** Claudia Hanson, Samiksha Singh, Karen Zamboni, Mukta Tyagi, Swecha Chamarty, Rajan Shukla, Joanna Schellenberg

**Affiliations:** 1 Department of Disease Control, London School of Hygiene and Tropical Medicine, London, England; 2 Department of Public Health Sciences, Karolinska Institutet, Stockholm, Sweden; 3 Public Health Foundation, India, Kavuri Hills, Madhapur, Hyderabad, India; International Institute for Population Sciences, INDIA

## Abstract

**Background:**

The Indian government supports both public- and private-sector provision of hospital care for neonates: neonatal intensive care is offered in public facilities alongside a rising number of private-for-profit providers. However, there are few published reports about mortality levels and care practices in these facilities. We aimed to assess care practices, causes of admission, and outcomes from neonatal intensive care units (NICUs) in public secondary and private tertiary hospitals and both public and private medical colleges enrolled in a quality improvement collaborative in Telangana and Andhra Pradesh—2 Indian states with a respective population of 35 and 50 million.

**Methods and findings:**

We conducted a cross-sectional study between 30 May and 26 August 2016 as part of a baseline evaluation in 52 consenting hospitals (26 public secondary hospitals, 5 public medical colleges, 15 private tertiary hospitals, and 6 private medical colleges) offering neonatal intensive care. We assessed the availability of staff and services, adherence to evidence-based practices at admission, and case fatality after admission to the NICU using a range of tools, including facility assessment, observations of admission, and abstraction of registers and telephone interviews after discharge. Our analysis is adjusted for clustering and weighted for caseload at the hospital level and presents findings stratified by type and ownership of hospitals. In total, the NICUs included just over 3,000 admissions per month. Staffing and infrastructure provision were largely according to government guidelines, except that only a mean of 1 but not the recommended 4 paediatricians were working in public secondary NICUs per 10 beds. On admission, all neonates admitted to private hospitals had auscultation (100%, 19 of 19 observations) but only 42% (95% confidence interval [CI] 25%–62%, *p*-value for difference is 0.361) in public secondary hospitals. The most common single cause of admission was preterm birth (25%) followed by jaundice (23%). Case-fatality rates at age 28 days after admission to a NICU were 4% (95% CI 2%–8%), 15% (9%–24%), 4% (2%–8%) and 2% (1%–5%) (Chi-squared *p* = 0.001) in public secondary hospitals, public medical colleges, private tertiary hospitals, and private medical colleges, respectively, according to facility registers. Case fatality according to postdischarge telephone interviews found rates of 12% (95% CI 7%–18%) for public secondary hospitals. Roughly 6% of admitted neonates were referred to another facility. Outcome data were missing for 27% and 8% of admissions to private tertiary hospitals and private medical colleges. Our study faced the limitation of missing data due to incomplete documentation. Further generalizability was limited due to the small sample size among private facilities.

**Conclusions:**

Our findings suggest differences in quality of neonatal intensive care and 28-day survival between the different types of hospitals, although comparison of outcomes is complicated by differences in the case mix and referral practices between hospitals. Uniform reporting of outcomes and risk factors across the private and public sectors is required to assess the benefits for the population of mixed-care provision.

## Abstract 2

Claudia Hanson and colleagues, part of the High Quality Care Commission assess care and outcomes in hospitals for neonates in 2 large states in India

## Author summary

**Why was this study done?:**

- The Indian government supports provision of neonatal intensive care for neonates in both public and private hospitals.
- High mortality after admission to public neonatal intensive care units (NICUs) has been described from facilities in India.
- Although it is often assumed that care is better in private rather than public facilities, studies in India and elsewhere do not provide a clear answer on this.

**What did the researchers do and find?:**

- We compared 26 public secondary hospitals, 5 public medical colleges, 15 private tertiary hospitals, and 6 private medical colleges, which together constitute roughly two-thirds of NICUs in Telangana and Andhra Pradesh, 2 Indian states with a respective population of 35 and 50 million.
- Equipment, supplies, and staffing were according to government guidelines except that rooms allowing mothers to breastfeed or to lie down for Kangaroo Mother Care were not available in all hospitals. Also, government hospitals had only 1 instead of 4 paediatricians per 10 beds as recommended in government guidelines. Admission care processes were substandard in relation to auscultation and temperature measurement, particularly in public facilities.
- Analysing 6,859 register records, we observed that 4%, 15%, 4%, and 2% of neonates admitted in public secondary hospitals, public medical colleges, private tertiary hospitals, and private medical colleges, respectively, died by the age of 28 days. However, analysis was complicated by a large number of missing records, which was more common in private than public facilities.

**What do these findings mean?:**

- The survival of neonates admitted to NICUs is better than previously described in Telangana and Andhra Pradesh.
- Comparison between private and public facilities is complicated by missing information both on outcomes and patient characteristics such as gestational age.
- Improved documentation and standardisation of registers in both the private and public sectors is essential for comparison between public and private hospitals.

## Introduction

The neonatal period—the first 28 days of life—is the most vulnerable period of childhood, and almost half of all neonatal deaths are in the first 24 hours of life. With improvements in basic neonatal care such as thermoregulation and breastfeeding, the provision of advanced care for neonates in need of hospital care is increasingly important for the reduction of neonatal mortality in low- and middle-income countries [1].

It is estimated that 605,000 neonates die in India each year with a neonatal mortality rate reported at 24 per 1,000 in 2018 [2]. In the past 15 years, the Indian government has made major investments to reduce both maternal and neonatal mortality by increasing accessibility to both childbirth and neonatal care. Several national programmes were launched to support facility delivery in public and private (in absence of public) facilities [3,4], provide free access to childbirth care and referral [5–7], and to provide early identification of and intervention for birth defects, diseases, and development disorders [8] (S1 Table). The India Newborn Action Plan, launched in 2014, aims to strengthen immediate and equitable newborn care, including care for small and sick newborns, and explicitly recommends involvement of the private sector under a joint regulatory framework [9]. To improve quality of facility delivery, 2 further programmes were launched in 2015 (DAKSHATA) and 2017 (LaQshya) [10,11]. The LaQshya initiative includes certification as part of National Quality Assurance Standards for both labour rooms and neonatal intensive care units (NICUs) in the public sector [12], whereas the National Neonatal Forum promotes and provides accreditation of newborn care units in the public and private sector [13].

Neonatal intensive care constitutes Level II care in special newborn care units (SNCUs) at district and subdistrict hospitals providing all types of care to newborns except surgery and Level III NICUs that provide intensive care including ventilation and operative care [14]. These neonatal intensive care facilities are complemented by Level I newborn stabilization units (NBSUs), which provide management of low birth weight (LBW) neonates not requiring intensive care, as well as stabilization of newborns before referral—and newborn care corners (NBCCs), which have been established at all facilities providing childbirth care, providing essential care at birth, including resuscitation [15–17]. The private sector offers a wide range of neonatal services, but there is no mapping of the newborn services in private hospitals. Some private hospitals offer neonatal intensive care at Level II, and a few offer Level III care [17].

The policies to improve childbirth and neonatal care have led to a large increase in the number of women delivering in health facilities in India (39% in 2005 to 2006 to 79% in 2015 to 2016) [18]. In the states of Telangana and Andhra Pradesh, more than 90% of births are in health facilities, of which at least half are in private facilities [18]. Between 2013 and 2015, about 1.23 million newborns were admitted to 525 SNCUs across India. Of these, 39% were outborn (born in another place and referred). Overall, 51% of the admitted neonates were underweight, and 44% had a gestational age of 36 weeks or less [19]. Very little is known, however, about NICU admissions in the private sector [17]. A study from rural Uttar Pradesh reported that more families of male neonates sought care at private facilities compared with families of female neonates [20].

There is considerable debate and conflicting evidence about whether the private or public sector provides better quality care in low- and middle- income countries [21,22]. Although the private sector includes a very diverse set of care providers and services, few studies have adjusted for facility factors such as size and type [23]. Also, few studies are available from India on this topic despite the large market share of the private sector and the fact that the majority of doctors work in the private sector [24,25], whereas more is known from the public sector [26]. One study from Uttar Pradesh, India, including a diverse mix of community health centres, district hospitals, and medical colleges, reported that quality of routine care for uncomplicated deliveries was generally poor but was better in the private compared with the public sector [27]. Another study indicated that knowledge of evidence-based practices seemed to be insufficient in practitioners from both the private and public sector in India [28].

In view of the public–private mix, which the Indian government supports to improve access to quality maternal and newborn care [9], and ongoing discussions on how to regulate and include the private sector effectively [29], we aimed to describe care practices, causes of admission, and neonatal outcomes from public and private NICUs that were later enrolled in a quality improvement collaborative in Telangana and Andhra Pradesh.

## Methods

This cross-sectional study uses data from 52 consenting hospitals from Andhra Pradesh and Telangana that were included in the baseline evaluation (30 May to 26 August 2016) of the Safe Care Saving Lives quality improvement collaborative programme, implemented by Access Health International, an international nongovernmental organisation. The evaluation was carried out independently by the Public Health Foundation of India together with London School of Hygiene and Tropical Medicine, UK.

### Study setting

Andhra Pradesh and the new state of Telangana, which was formed in 2014 when Andhra Pradesh split into 2, are situated in southern India and are characterised by slightly better socio-economic development indicators than the Indian average [30]. In 2014 to 2015, the sex ratio among children under 5 years old was 1,000 to 890 in both states. Most mothers (97% in urban and 91% in rural areas) delivered in health facilities, but less than 34% in urban and 39% in rural areas delivered in public health facilities [31,32]. However, because specialised newborn care is more expensive in the private sector, most seek public healthcare for very sick newborns [17]. The neonatal mortality rate was estimated, in 2016, at 11 and 15 per 1,000 live births in urban areas of Andhra Pradesh and Telangana and 27 and 25 per 1,000 livebirths in rural areas of Andhra Pradesh and Telangana [33]. As in other parts of India, the main causes of neonatal death are prematurity, LBW, and intrapartum-related complications (also known as birth asphyxia) together with neonatal infection [34].

### Study design

The evaluation protocol is described in detail elsewhere [35]. Here, we use the Strengthening the Reporting of Observational Studies in Epidemiology (STROBE) checklist for reporting (S1 STROBE Checklist). In short, the intervention targeted care provision in NICUs of Level II (SNCU) and III (NICU), as well as labour wards of public and private hospitals that were enrolled in a government-funded Health Care Trust. The quality improvement approach was modelled on the Institute of Health Care Improvement’s collaborative quality improvement approach in which teams in different institutions work on similar problems [36]. The intervention was supporting the consistent implementation of evidence-based care in these NICUs. The quality improvement programme was organised in 3 phases to cover all 85 public and private hospitals that provided neonatal intensive care in the 2 states. Phase I included 25 self-selected hospitals that were not included in the evaluation. Phase II and III together comprised the remaining 60 hospitals [37]. The NICUs included here are just over two-thirds of all such units in the 2 states of Andhra Pradesh and Telangana, together serving a population of 85 million people. Here, we use cross-sectional data from the baseline assessment conducted before a trial (http://ctri.nic.in/Clinicaltrials/showallp.php?mid1=19367&EncHid=&userName=Samiksha%20Singh).

### Inclusion of participants

**Fig 1:**
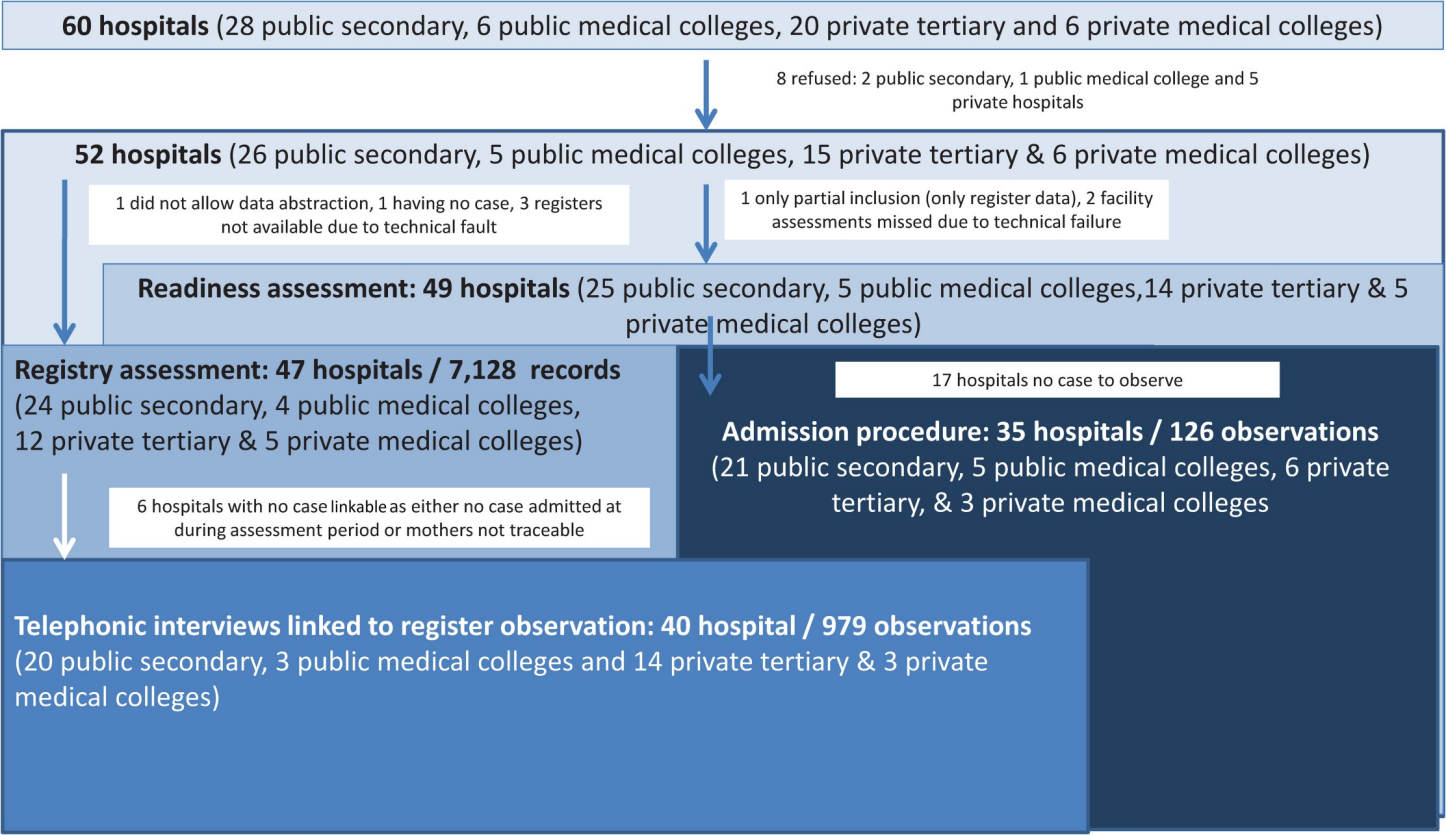
Inclusion of participants.

Our study targeted 60 secondary and tertiary hospitals that were to be included in the evaluation of the quality improvement intervention. The public facilities comprised 12 district hospitals, 10 area hospitals, 4 maternal and child health hospitals, which are classified together here as public secondary hospitals, and 5 public medical colleges. The private hospitals comprised 11 speciality hospitals, 5 superspeciality hospitals, which are classified together here as private tertiary, and 6 medical colleges, providing care at Level III (Fig 1).

### Data collection process and tools

Data were collected by a team of study data collectors (nurses or public health specialists) who visited each hospital for a period of 5 days. Data collectors were remunerated based on standard salary schemes. They were trained for 4 days, followed by pilot data collection and by 2 more rounds of re-training of 3 days each. We included (i) a facility checklist, (ii) observation of the admissions procedure to the NICU, (iii) an abstraction of NICUs register, and (iv) postdischarge telephone interviews with mothers whose neonates had been admitted to the NICU (S1 Fig).

The facility checklists were based on standards for labour room and newborn care services in India [38,39]. All neonates who were admitted to the NICU were observed (during day and night on alternate days) by trained nurses who used a checklist to document the admission services provided. Using a data abstraction form, we intended to abstract either the 200 most recent admissions in each hospital or all admissions in the last 3 months, whichever was less. We abstracted these admission data from paper-based registers at each hospital. These registers were standardised in the public sector and were also entered in an electronic database supported by UNICEF [19]. In the private sector, there was a larger variation in the format of these paper-based registers. Almost all of these registers also recorded telephone numbers of mothers and families of neonates admitted to the NICU. We abstracted the respective telephone numbers together with the information from the registers for the last 1 month in each of the hospitals. Four public health graduates or nurses specifically trained on interviewing skills for 1 day conducted interviews over the phone using a structured interview guideline. We obtained the outcome of the neonates on the 7th and 28th day of life. In a hospital with a case load of less than 50 neonates in the last month, we called all numbers available. If more than 50 contact numbers were available, we used systematic sampling to select 50 at random. Those neonates that were more than 28 days old at admission were excluded.

Data collection was organised in teams composed of 1 supervisor, 1 data abstractor, 2 observers, and 1 interviewer. We formed a total of 6 teams. All tools were programmed using a native and SQLite application and linked with a backend server database. Android-based tablets (Lenovo, TAB 2 A7-20, Leveno PC HK Limited, China) were used for data collection and upload. The application used skips and ranges to improve data quality. Four public health or graduate nurses trained in interviewing skills were responsible for telephone interviews. Data were saved daily and uploaded on a secure server weekly. The data were checked on a weekly basis.

### Key outcome measures

Our measures of interest included admission diagnosis abstracted from the registers and neonatal mortality (case-fatality rate) at 7 and 28 days of life among those admitted in the newborn intensive care units. We also included measures of processes (admission procedures) and inputs (staffing and availability of services). We grouped the admission diagnosis into 5 key groups of premature or LBW, asphyxia, sepsis, jaundice, and others (other than the former 4 including malformations, etc.). As many neonates had more than one admission diagnosis, we treated premature or LBW as the most important diagnosis, followed by asphyxia, sepsis, and jaundice. We also tabulated the overlap between diagnosis groups. We assessed case fatality after admission to a NICU and computed 7-day and 28-day-of-life case-fatality rates after admission to a NICU to reflect the higher vulnerability of the neonates during the first 7 days of life compared with 7 to 28 days of life. We merged the data set abstracting the register information and telephone interviews to assess mortality after discharge or referral.

### Analysis

Analysis was done in Stata 13 (StataCorp, Texas). We grouped the hospitals into 4 categories: (i) public secondary hospitals, including district, area, and maternal and child hospitals, (ii) public medical colleges, (iii) private tertiary hospitals, and (iv) private medical colleges reflecting the levels of neonatal intensive care. We weighted the analysis of (i) the admission observations and (ii) the register-based outcomes after admission by average caseload for the hospital as assessed during the facility assessment. Furthermore, we adjusted the estimates for clustering at the hospital level using svy commands. We tabulated frequencies and 95% confidence intervals (CIs) of estimates. We faced a large number of missing variables for newborn outcomes as well as for important risk factors (S2 Table). We assessed case fatality excluding neonates admitted only because of jaundice because this complication, strictly speaking, does not warrant admission to the NICU. Information on gestational age and birth weight was available for only 4,078 register observations (60%). We conducted adjusted analysis for this subset to account for gestational age and birth weight, which are major predictors of case fatality. Because of the large amounts of missing data and the biases that this may introduce, we present only descriptive statistics.

### Ethics and consent

Ethical approval was granted from LSHTM (LSHTM Ethics Reference 10358) and PHFI’s Institutional Ethics Committee (IIPHH/TRCIEC/064/2015). The study complies with the International Ethical Guidelines for Biomedical Research Involving Human Subjects and the principles of the declaration of Helsinki [40]. An information sheet was read out to each participant. Written consent was obtained from each participating hospital, and verbal consent was obtained from health providers and mothers. Participants could withdraw at any time. Confidentiality was assured, as per institutional guidelines of both involved institutions.

## Results

Of the 60 hospitals contacted for the study (28 public secondary, 6 public medical colleges, 20 private tertiary, and 6 private medical colleges), the facility heads from 8 refused participation of their hospital (2 public secondary hospitals, 1 medical college, and 5 private tertiary; Fig 1). Facility readiness assessments were conducted in 49 hospitals (25 public secondary, 5 public colleges, 14 tertiary hospitals, and 5 private medical colleges), and observation of admission to the NICU was conducted in 35 hospitals (21 public secondary, 5 private colleges, 6 private tertiary hospitals, and 3 private medical colleges). The assessment of register data was available from 47 hospitals (24 public secondary, 4 public medical colleges, 14 private tertiary hospitals, and 5 private medical colleges). Information from a total of 979 telephone interviews with mothers (54% of intended interviews) were linked with their newborns’ outcome information from the registers to estimate overall outcome including postdischarge period.

Whereas only 9 of the 19 consenting private hospitals and medical colleges had a labour room (Table 1), all 5 public medical colleges had one. All 28 public secondary hospitals with a NICU also offered delivery care. In total, the NICUs had just over 3,000 admissions per month. The median number of monthly admissions to the NICUs was 82 (interquartile range [IQR] 55–138), 148 (IQR 110–176), 30 (IQR 21–45), and 47 (IQR 38–53) in public secondary hospitals, public medical colleges, private tertiary hospitals, and private medical colleges, respectively. The median number of monthly admissions per available bed in the NICU was 4.4, 8.4, 1.4, and 3.9 in public secondary hospitals, public medical colleges, private tertiary hospitals, and private medical colleges, respectively.

**Table 1:** General information by type of hospital.

	Public secondary		Public medical college		Private tertiary		Private medical college		Explanation of those missing
	*N* = 28		*N* = 6		*N* = 20		*N* = 6		
	*N* or mean/median	% or IQR	*N* or mean/median	% or IQR	*N* or mean/median	% or IQR	N or mean /median	% or IQR	
***Agreed participation in baseline assessment (full or partial)***	26		5		15		6		
***Labour room assessment***	26		4		3		6		12 private hospitals had no labour ward, 1 public secondary hospital refused data collection, and 1 public medical college missing
***No. of deliveries per month***	282/200	99–382	584/472	324–845	24/29	11–32	136/167	39/195	
***Neonatal care unit assessment***	25		5		14		5		
***Breastfeeding room***	21	87%	4	80%	11	85%	5	100%	1 public secondary and 1 private tertiary
***Kangaroo Mother care room***^***#***^	14	70%	3	60%	7	50%	2	40%	5 public secondary
***No. of admissions per month***	91/82	55–138	143/148	110–176	31/30	21–45	47/47	38–53	3 public secondary, 1 public medical college, 1 private tertiary, and 1 private medical college
***Beds in neonatal care unit***	18/18	14–20	18/18	18–20	17/18	12–20	12/12	10–14	1 public secondary, 1 private tertiary
***Monthly admission to bed ratio***	5.5 /4.4	3.4–5.9	8.8/8.4	5.4–12.2	1.9/1.4	1.2–2.5	4/3.9	2.6–5.3	3 public secondary, 1 public medical college, 2 private tertiary, 1 private medical college
***No. of paediatricians***	3/2	1–3	4/4	2–6	6/4	2–6	7/7	6–7	1 public secondary, 1 private tertiary
***No. of nurses***	10/11	6–13	15/14	14–18	8/8	3–12	14/11	10–14	
***Paediatricians per 10 beds***	1/1	1–2	2/2	1–3	4/3	1–3	6/6	5–7	
***Nurses per 10 beds***	6/6	4–7	9/8	7–10	6/6	2–8	13/10	7–14	

^#^A Kangaroo Mother Care room allows the mother to have a bed or comfortable chair to keep her baby on the chest.

**Abbreviation:** IQR, interquartile range

Most hospitals had a breastfeeding room: 87%, 80%, 85%, and 100% of public secondary, public medical colleges, private tertiary, and private medical colleges, respectively. The median number of paediatricians per hospital was 2, 4, 4, and 7 in public secondary, public medical colleges, private tertiary, and private medical colleges, with differences becoming more marked when expressed as the ratio of paediatricians per 10 beds: a median of 6 paediatricians for 10 beds in the private medical colleges compared with a median of 1 paediatrician in public secondary hospitals.

We observed 126 admissions to a NICU (81 in public secondary hospitals, 26 in public medical colleges, 12 in private tertiary hospitals, and 7 in private medical colleges). Amongst those observed, 48% of public secondary and 78% of public medical colleges’ admission were done by a medical doctor without further specialisation. Admissions in private tertiary hospitals were done in 49% of cases by a nurse and in 45% of cases by a paediatrician. All observed admissions in private medical colleges were done by a paediatrician (Table 2). Although the neonate’s history was taken in almost all admissions, auscultation was universal in private hospitals yet was done for 42% (95% CI 25%–62%) in public secondary hospitals and 47% (95% CI 13%–84%, chi-squared adjusted for clustering *p* = 0.361) in public medical colleges. Appropriate hand hygiene before examination was observed in 100% of private tertiary and in 76% of private medical college, but in only 40% in public secondary and 31% in public medical colleges (*p* = 0.056).

**Table 2:** Care at admission to neonatal care unit by type of hospital.

		Public secondary^a^ % (95% CI)	Public medical college^b^ % (95% CI)	Private tertiary^c^ % (95% CI)	Private medical college^d^ % (95% CI)	P value (*c*hi-squared test) adjusted for clustering
**Admission staff**^**e**^	**Paediatrician**	21 (10–39)	14 (5–35)	45 (29–61)	100	0.045
	**Medical doctor**	48 (25–73)	78 (47–93)	6 (1–40)	0	
	**Nurse**	31 (17–48)	8 (1–38)	49 (32–67)	0	
**History taken**^**f**^		92 (77–98)	92 (67–99)	89 (44–99)	100	0.953
**Auscultation done**^**g**^		42 (25–62)	47 (13–84)	100	100	0.361
**Temperature taken**^**h**^		30 (12–56)	27 (9–57)	59 (14–93)	100	0.218
**Neonate weighed**		87 (75–94)	97 (83–99)	100	100	0.500
**Hand hygiene before examination**^**i**^		40 (29–52)	31 (15–52)	100	76 (26–97)	0.056
**Sent back or referred**^**j**^		9 (3–26)	0	0	0	0.593

All estimates weighted according to average case load in neonatal care unit in the 3 months before the observations.

^a^21 hospitals; 81 observations included

^b^5 hospitals; 26 observations included

^c^6 hospitals; 12 observations included

^d^3 hospitals; 7 observations included

^e^2 missing values from 1 public secondary

^f^3 missing values from 1 public secondary

^g^1 missing from 1 public secondary

^h^1 missing from public secondary, 1 public medical college

^i^1 missing from public secondary and 1 from private tertiary

^j^1 missing from public secondary

**Abbreviation:** CI, confidence interval

**Fig 2:**
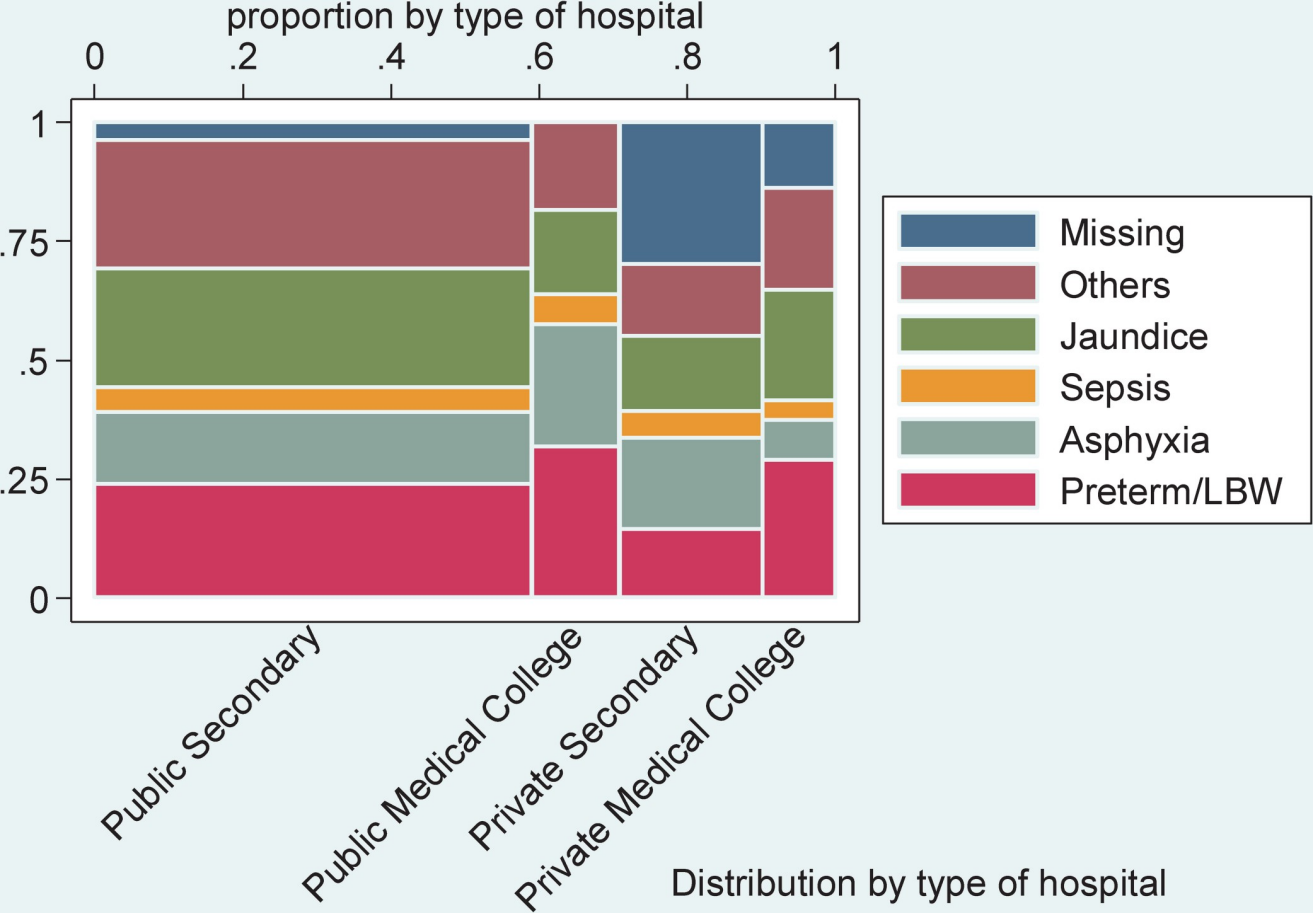
Admission diagnosis by type of hospital based on register data. LAMA, left against medical advice.

Of the 7,128 abstracted records, 269 records were excluded from the analysis because the neonates were still admitted, and thus no final outcome was available. Of the 6,859 included neonatal admissions, the most common admission diagnosis was prematurity or LBW (24.1% of admissions), followed by jaundice (22.9%), asphyxia (16.3%), and sepsis (5.4%) (Fig 2). Of note, in public medical colleges, all neonates had an admission diagnosis, whereas in public secondary and private tertiary hospitals, the admission diagnosis was missing for 4.9% and 25.7% of neonates, respectively.

**Fig 3:**
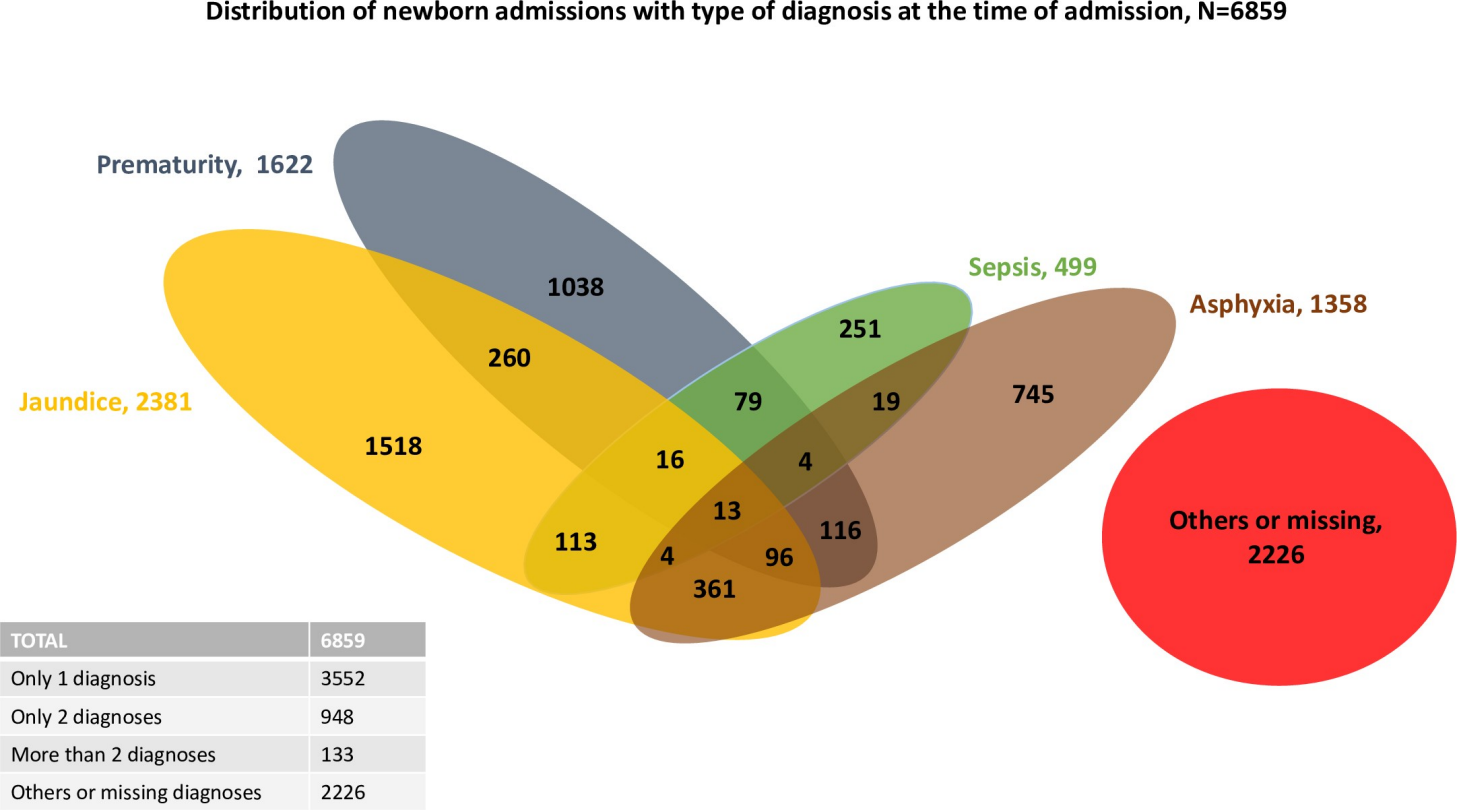
Admission diagnosis and overlap (not to scale) based on register data. Note: others included, for example, malformation, pertussis infection, etc.

Fifty-two percent (3,552 of 6,859; 51.7%) of admitted neonates had 1 admission diagnosis, whereas 948 of the 6,859 (13.8%) had 2 diagnoses, most commonly asphyxia or prematurity combined with jaundice (Fig 3).

**Fig 4:**
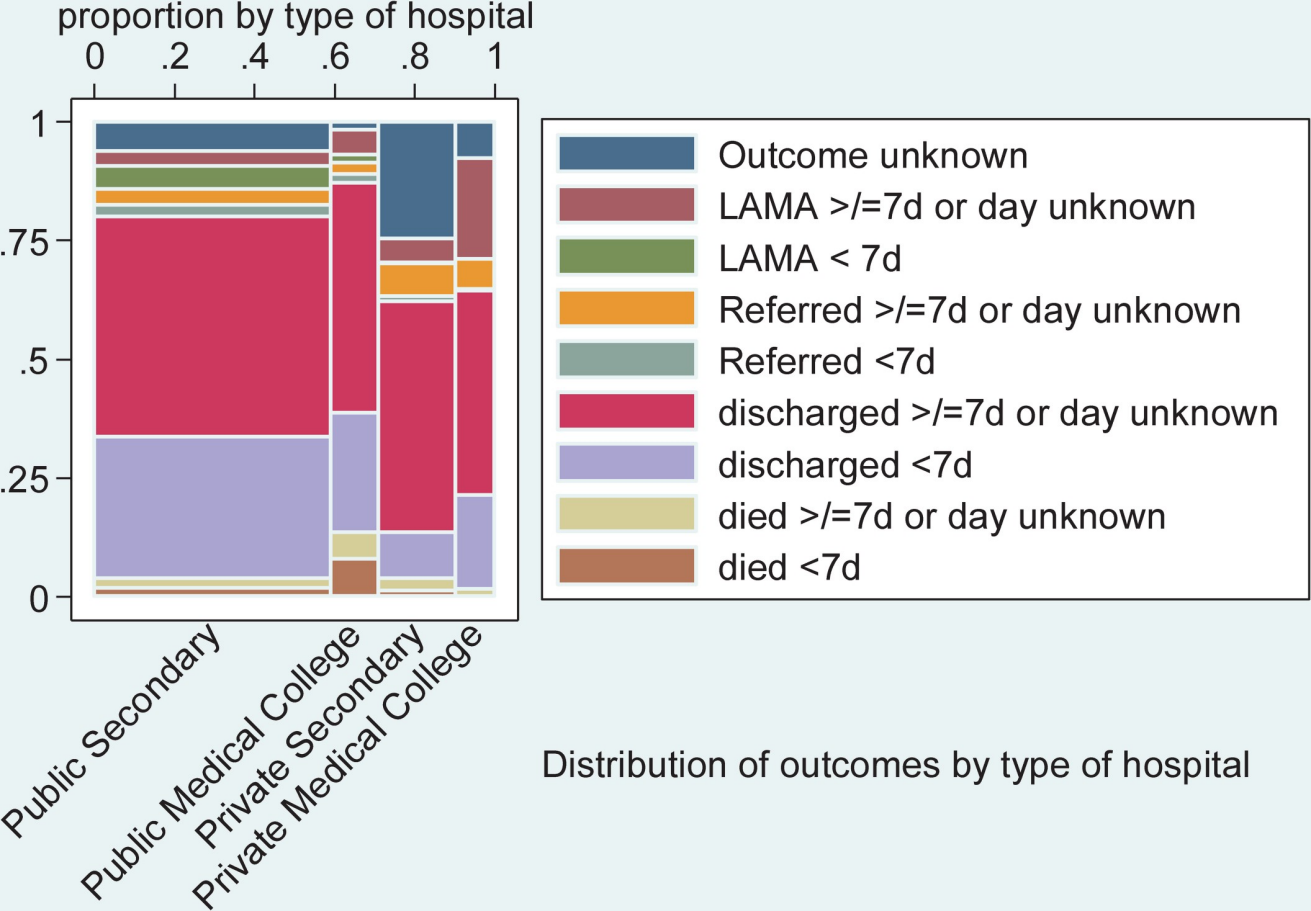
Babies’ outcome after admission to neonatal care unit by type of hospital based on register data. LAMA, left against medical advice; LBW, low birth weight.

Three-quarters of all neonates admitted to the NICU were either discharged within the first 7 days of life (27.5%) or 7 to 28 days after birth (45.0%), with some variation between the types of hospitals (Fig 4 and S2 Table). Almost 6% of neonates were referred to other facilities (information on place of referral not available; 2.2% within the first 7 days of life and 3.6% in 7 to 28 days of life); 3.5% of parents left with their neonates against medical advice during the first 7 days, and a further 4.3% left between 7 and 28 days of life. In private hospitals, the outcome was not documented for 20.2% of admitted neonates.

Overall, 7-day case fatality was highest in public medical colleges, and particularly high in neonates admitted because of prematurity or LBW (17.3%; 95% CI 3.7%–53.2%, *p* < 0.001 for difference in mortality by diagnoses; Table 3). In contrast, private tertiary hospitals’ NICUs had a lower 7-day case fatality in neonates admitted because of prematurity of only 1.7% (95% CI 0.5%–4.9%). Because no information was available on the date of birth from private medical colleges’ registers, 7-day mortality could not be calculated.

**Table 3:** Case fatality by admission diagnosis based on register data by type of hospital.

		Public secondary^a^		Public medical college^b^		Private tertiary^c^		Private medical college^d^		Overall^e^	
	Number	% (95% CI)									
		<7 days	7–28 days or day unknown^f^	<7 days	7–28 days or day unknown^g^	<7 days	7–28 days or day unknown^h^	<7 days	7–28 days or day unknown^i^	<7 days	7–28 days or day unknown
***Preterm/LBW***	1,611	4.8 (2.5–9.0)	6.7 (1.8–21.4)	17.3 (3.7–53.2)	9.4 (2.0–34.1)	1.7 (0.5–4.9)	3.6 (0.9–14.1)	0	2.6 (0.6–10.5)	7.2 (4–0–12.8)	6.9 (3.1–14.6)
***Asphyxia***	1,126	2.8 (1.3–5.8)	0.9 (0.4–2.5)	9.2 (0.4–70.0)	5.8 (0.6–38.4)	0.5 (0.1–5.2)	2.3 (1.0–5.2)	0	6.8 (3.6–12.5)	4.1 (1.8–9.0)	2.5 (1.1–5.5)
***Sepsis***	362	0.4 (0.1–2.8)	2.9 (1.0–7.9)	9.1 (0.8–54.3)	2.2 (0–54.0)	0.6 (0.1–6.7)	1.7 (0.2–16.9)	0	0	2.4 (0.7–8.1)	2.5 (1.1–5.4)
***Jaundice***	1,513	0.8 (0.2–2.7)	0	0.6 (0–11.5)	1.1 (0.1–12.2)	0	1.6 (0.4–5.8)	0	0	0.6 (0.2–1.9)	0.3 (0.1–0.8)
***Others***	1,570	0.6 (0.1–2.4)	1.0 (0.2–4.5)	4.6 (0.4–35.5)	2.3 (0.6–8.9)	1.3 (0.2–10.1)	0.8 (0.2–3.8)	0	0.7 (0.1–6.1)	1.2 (0.5–2.7)	1.1 (0.4–3.0)
***Missing***	638	2.1 (0.6–7.6)	3.5 (0.9–12.6)	-	-	1.5 (1.2–1.8)	5.8 (4.0–8.3)	0	0.8 (0–19.2)	1.6 (0.7–3.7)	4.1 (2.1–8.0)
***Overall***	6,820	2.0 (1.0–4.0)	2.3 (0.7–7.5)	9.6 (1.3–46.6)	5.2 (1.8–14.5)	01.0 (0.4–2.5)	2.6 (1.2–5.4)	0	1.5 (0.2–8.8)	3.1 (1.6–5.9)	2.8 (1.4–5.6)
***Overall excluding jaundice***	5,307	2.4 (1.2–4.6)	3.1 (0.9–9.6)	11.5 (5.3–23.2)	6.1 (3.8–9.8)	1.2 (0.5–2.8)	3.5 (1.8–6.8)	0	2.0 (0.7–5.6)	3.8 (1.9–7.4)	3.6 (1.8–7.1)

^a^24 hospitals observed; 4,027 case fatalities; 248 observations missing; *p* < 0.020 for difference between type of diagnosis

^b^4 hospital observed; 807 case fatalities; 13 observations missing; *p* < 0.057 for difference between type of diagnosis

^c^14 hospitals observed; 1,314 case fatalities; 255 observations missing; *p* < 0.173 for difference between type of diagnosis

^d^5 hospitals observed; 672 case fatalities; 122 observations missing; *p* < 0.062 for difference between type of diagnosis

^e^6,820 admission diagnoses; *p* < 0.001 for difference between type of diagnosis

^f^The age of the newborn at death could not be calculated for 1,848 (55%) observations because the date of birth of the neonate was not documented

^g^The age of the newborn at death could not be calculated for 314 (39%) observations because the date of birth of the neonate was not documented

^h^The age of the newborn at death could not be calculated for1,028 (78%) observations because the date of birth of the neonate was not documented

^i^The age of the newborn at death could not be calculated for 458 (68%) observations because the date of birth of the neonate was not documented

**Abbreviations:** CI, confidence interval; LBW, low birth weight

The subgroup analysis of 4,078 of 6,820 records with gestational age or birth weight available suggested very similar admission patterns in relation to birth weight across the 4 types of hospitals; 30% of babies were premature or of LBW. Public medical colleges reported a high case fatality of 31.3% (95% CI 13.4%–47.4%) in neonates of 32 to 36 weeks of gestational age (S3 Table).

We observed a 7-day case fatality of 2.7% (95% CI 1.4%–5.5) and 4.4% (95% CI 2.3%–8.2%) and a 7-to-28-day case fatality of 1.8% (95% CI 0.7%–4.1%) and 4.9% (95% CI 2.7%–8.7%) in inborn (born in the same hospital) and outborn neonates (born outside the study hospital and referred), respectively, combining all observations in all NICUs (S2 Fig).

We linked 979 mother interviews with the register data of their babies while admitted to a NICU. Although the register data suggested an overall case fatality within the first 28 days of life after admission to the NICU of 5.9% (95% CI 3.6%–9.7%), the estimate was substantially higher at 11.6% (8.1%–16.3%) when we included deaths reported by mothers during the telephone interviews (Table 4). The largest number of additional deaths reported by mothers were in cases in which the outcome was missing in the registers or the newborn was discharged against medical advice from public secondary facilities. The telephone interviews suggested that 36.6% of the neonates discharged against medical advice died, as did 8.1% of neonates for which no outcome was documented (S4 Table).

**Table 4:** Twenty-eight-day case fatality of babies after admission to neonatal care unit by register data and telephone interviews and by type of hospital.

	28-day case fatality according to 6,820 register observations % (95% CI) p = 0.0009	No. hospitals/no. observations^#^	Deaths reported in subset of 979 neonates admitted to the NICU and followed by telephone interview up to 28 days of life						28-day case fatality according to telephone interviews	
			Register-reported deaths	Discharged	Referred	LAMA	Outcome unknown	Total	Total admissions (*N*)	% (95% CI)
**Public secondary**	4.3% (2.1%–8.4%)	20/618	32	5	1	12	19	69	618	11.5% (7.2–17.9%)
**Public medical college**	14.8% (8.8%–23.9%)	3/123	3	2	0	1	13	19	123	16.4% (12.0–22.0%)
**Private secondary**	4.2% (2.3%–7.6%)	14/215	1	1	0	2	9	13	215	5.4% (2.1–13.0%)
**Private medical college**	1.5% (0.5%–4.9%)	3/23	1	0	0	0	0	1	23	4.2% (0.4–31.1%)
**All hospitals**	5.9% (3.6%–9.7%)	40/979	39	8	1	15	41	102	979	11.6% (8.1–16.3%)

^#^In 4 public secondary, 1 public medical college, and 2 private medical college, no case could be linked interviews because no case was admitted or mothers were not traceable.

**Abbreviations:** CI, confidence interval; LAMA, left against medical advice; NICU, neonatal intensive care unit

## Discussion

Our study spanned inputs, processes, and case fatality at 7 and 28 days of age, comparing public secondary hospitals, public medical colleges, and private hospitals and private medical colleges and indicates suboptimal and a high 28-day case fatality of 5.9% after admission to a NICU. Although prematurity is, as expected, the most common admission diagnosis (25% of admissions), we were surprised to find that jaundice was the second most common, accounting for 23% of admissions. Case fatality was highest in medical colleges, particularly for prematurely born neonates, of whom 25% died within the first 28 days of life. Our telephone interviews with mothers whose newborn was admitted to a NICU suggest that hospital registers substantially underestimate the 28-day case fatality. Whereas 28-day case fatality was 5.9% (95% CI 3.6%–9.7%) across registers from the 4 types of hospitals, case fatality was 11.6% (95% CI 8.1%–16.3%) according to postdischarge telephone interviews. Differences may be explained both by registers being incomplete and by a high mortality in neonates discharged against medical advice.

### Infrastructure and neonatal intensive care practices

We report 6 and 8 nurses per 10 beds in public secondary hospitals and medical colleges, respectively, which correspond to the staffing standards as laid down in the Indian Newborn Action Plan [9]. In contrast, staffing ratios for paediatricians were only met in public medical colleges and the private hospitals. Although the staffing norms for nurses were met, we cannot conclude that the ratio is adequate in relation to caseload, bed occupancy rates, and the case mix [41]. Negative mortality impact of workload has been reported in a study from the United Kingdom [42]. We found that specialist doctors were more commonly available in private than public hospitals, a finding that has also been described in regard to emergency obstetric care in India [43]. Despite this, admission rates are higher in the public sector, which serve low-income groups and cannot refuse admission despite overcrowding. Similarly, a review comparing private and public sector quality of care in low- and middle-income countries indicated better staffing in private compared with public facilities [22].

We found that auscultation of the newborn, temperature measurement, and hand hygiene was more commonly implemented in private tertiary hospital or private medical colleges than the public facilities, although our estimates were imprecise due to the small sample. Similarly, Sharma and colleagues indicated better adherence to evidence-based practices for routine childbirth care in private compared with public hospitals in Uttar Pradesh, India [27]. Also, the review by Berendes comparing ambulatory care in private and public facilities in low- and middle-income countries indicated marginally better adherence to good practice in the private compared with the public sector [21]. A study from Sri Lanka highlighted that the private sector performed better in those aspects in which resources were important to implement consistently [44]. Our finding that auscultation at admission was more consistently done in private compared with public facilities might be rooted in the fact that paediatricians were more consistently available to admit neonates. The review cited earlier proposed that adherence to guidelines is often worse in the private compared with the public sector, particularly when including potentially unnecessary interventions such as prescription of antibiotics or cesarean section [22]. We did not assess any indicator of overuse and cannot exclude the possibility that in our setting this aspect of quality might also be worse in private compared with public hospitals.

### Admission diagnosis and mortality

Our analysis found that 23% of admissions to NICUs were due to jaundice alone, regardless of the type of hospital. This is at odds with an earlier report from Andhra Pradesh (April 2013 to March 2015) that described only 14% of admissions being due to jaundice [19]. A recent review indicated that clinically significant jaundice is expected to occur in 10% of neonates and that jaundice is the leading cause of hospital admission of neonates worldwide, with the highest mortality rates in South Asia [45]. Nevertheless, it is expected that most cases can be managed with light therapy and have no need for intensive care, making the high rates of admission somewhat surprising [45].

### Case fatality

We describe case-fatality rates after admission to NICUs of 5.9%: 3.1% within the first 7 days and an additional 2.8% during days 7–28. Case fatality was, as expected, highest in neonates admitted because of prematurity. This is in line with other studies describing the continuous high burden of mortality in premature neonates in India [46]. Our observation that the temperature at admission—a most crucial quality step in premature babies—was not consistently checked gives a hint that substandard care may be responsible for the high case fatality.

Our case-fatality estimate is, however, lower than previously described. One of the first studies after introduction of SNCUs in India reported case-fatality rates of almost 30% within the first year of operation in 2003 in West Bengal [47]. A report from Andhra Pradesh (April 2013 to March 2015) described fatality rates of 10% in inborn and 15% in outborn neonates in the public SNCUs [19]. The high rate of admission for jaundice might bias the results, yet even when we excluded jaundice, our observed case fatality was only 7.4%. Mortality in our study, however, could also be higher as our postdischarge interviews point to a high mortality.

More than 5% of neonates admitted to a NICU were referred out, with particularly high rates from the private tertiary hospitals and private medical colleges. Private facilities may refer neonates to medical colleges because they may not be able to offer sufficiently specialised care or they may want to prevent newborn deaths in their records. Referral might also be on request of families, because costs for care of neonates are high in private facilities [48] and poor families might not be able to afford many days of treatment. The government-funded Health Care Trust only covers expenses for very sick neonates, for example, needing ventilation, whereas neonates not in need of such care are not included.

Neonatal intensive care register data from the public sector were relatively complete—probably because of the mandatory use of an electronic registry. In contrast, information completeness in the private sector was poor. Previous reviews have highlighted that the private sector lacks published data [22], and here, we describe that also the primary sources of data, the registers, are not well maintained. We had expected better data availability because the private hospitals were part of a state Health Care Trust [49], but the potential regulatory power on data availability and accountability is obviously not leveraged [50]. Data sharing between the private and public sectors has been also previously described as rather informal in India [51]. The power of hospital data to explain mortality patterns and risk factors is thus not leveraged despite its importance to inform the development of high-quality health systems and services [52]. Our study underlines the potential and importance of uniform electronic registries but also the limitations if the private sector is not obliged to use the standard reporting.

### Methodological considerations

Although we compared a number of dimensions of quality of care, including structural inputs, processes, and case fatality, we do not report on others such as user satisfaction and service efficiencies in which major differences between the public and private sector have been described [22]. Also, we were unable to report on technical quality of care to manage the neonates admitted to the hospitals, and thus it is difficult to establish a link between quality and case fatality. Our further results on inadequate hand hygiene are reported elsewhere [53]. Also, we could not assess whether performance of providers in the private sector could have been more effective because they deal with patients with both higher income and expectations of care.

We were only able to include a small sample for the results on admission procedures because the data collection team stayed only 5 days in each facility, which limits power and precision. The direct observations could also have been subject to social desirability bias. Also, we were only able to get in contact with just over 50% of mothers whose neonates were discharged from the neonatal ward. Despite this limitation, our analysis suggests that reported case-fatality rates might be biased downwards because of incomplete documentation and discharge practices. The small sample size in the private section limits, in particular, the generalisability of our findings in regard to the private sector.

Another important limitation is that the cause of mortality was not mentioned in any of the registers, and case-fatality rates thus relate to cause of admission only. We have no information about how well this diagnosis related to the true morbidity pattern the newborn presented at admission.

We had to deal with a large number of missing outcome data as well as the fact that important risk factor information was not available for a large number of records. Missing data also related to the date of birth, which constrains the possibility to calculate 7-day and 7–28-day case fatality. The missing risk factors limit the opportunity to predict mortality using severity markers as done elsewhere [54]. It also limits the possibility of estimating case fatality adjusted for important risk factors. This, in turn, severely constrains comparability across public and private tertiary hospitals and medical colleges, which is why we have not presented comparative statistics. In view of the large amount of missing outcomes and the high case fatality reported in the telephone interviews, such an analysis would give results that are hard to interpret. Large missing data in date of outcome limited us from computing duration of stay.

### Conclusion

We observed differences in 28-day survival by type of hospital. Case-fatality rates after admission were highest in public medical colleges, probably because they care for the sickest neonates. Twenty-three percent of admissions to NICUs were due to jaundice only, which is surprising. However, comparison of outcomes was severely constrained by differences in referral practices and recording completeness between private and public facilities. Uniform reliable reporting of outcomes and risk factors across all type and ownership of facilities is needed.

## Supporting information

S1 Table Main policies supporting access and quality to maternal and newborn care in India. (DOCX)

S1 STROBE Checklist STROBE, strengthening the reporting of observational studies in epidemiology. (DOCX)

S1 Fig Data collection flow. (TIF)

S2 Table Available risk factors and completeness of register observations. (DOCX)

S3 Table Outcomes after admission to NICU by type of hospital using register data. NICU, neonatal intensive care unit
(DOCX)

S2 Fig Distribution of outcomes by type of admission (inborn or outborn). (TIF)

S4 Table Babies’ outcome after admission to neonatal care unit assessed by telephonic follow-ups. (DOCX)

S1 Data Data collection tool: Labour room assessment. (DOCX)

S2 Data Data collection tool: NICU assessment. NICU, neonatal intensive care unit
(DOCX)

S3 Data Data collection tool: Observations. (DOCX)

S4 Data Data collection tool: Abstraction of registers. (DOCX)

S5 Data Data collection tool: Telephone interviews. (DOCX)

## Abbreviations

CI: confidence interval
IQR: interquartile range
LAMA: left against medical advice
LBW: low birth weight
NBSU: newborn stabilitization unit
NCC: newborn care corner
NICU: neonatal intensive care unit
SNCU: special newborn care unit
STROBE: Strengthening the Reporting of Observational Studies in Epidemiology
