# Supplementary material for: Care practices and neonatal survival in 52 neonatal intensive care units in Telangana and Andhra Pradesh, India: A cross-sectional study
Source: PLoS Med. 2019 Jul 23;16(7):e1002860. doi: 10.1371/journal.pmed.1002860 (PMC6650044; doi:10.1371/journal.pmed.1002860)
Supplement: S1 Table — (DOCX) [file pmed.1002860.s001.docx]

S1 Table: Main policies supporting access and quality to maternal and newborn care in India (references as in main body of text)

| **Policy /year of initiation** | **What it was expected to do** | **What it does** |
| --- | --- | --- |
| **Janani Suraksha Yojana (JSY), 2005** | Provides conditional cash transfers to women for delivery and post-delivery care to enable behavior change and increase institutional deliveries. The incentive differs across states (low or high performing states) and in rural or urban area (1, 2). | After the introduction of the policy a steep rise in facility births across the country was observed. Over 50% of women who had a previous home delivery opted for an institutional delivery; however, the programme did not improve access to skilled birth attendance (SBA) as most nurses and Auxiliary Nurse Midwifes were not sufficiently trained (1, 2). |
| **Janani Shishu Suraksha Karyakram, 2011** | Janani Shishu Suraksha Karyakram (JSSK) scheme was launched in 2011, to provide free and cashless delivery and services to sick newborns, so that no costs for drugs, consumables, diagnostics, diet, blood, transport for women to access childbirth care, referral or travel home were to be paid by families (3-5). | The scheme is widely used. However, a study conducted in rural areas of North India in 2017 found an underutilization of JSSK scheme due to lack of facilities in peripheral areas and therefore, still high expenses are made by the women. Referral transport is well utilized for delivery care and referral in pregnancy and intrapartum but less so for sick newborns (3-5) . |
| **Rashtriya Bal Swasthya Karyakram, 2013** | Rashtriya Bal Swasthya Karyakram (RBSK) aims for early identification of and intervention for defects at birth, deficiencies, diseases and development delays including disabilities, from birth to 18 years. Screening for defects at birth is envisaged as a role of facilities and community workers (6). | No assessment found. |
| **India Newborn Action Plan, 2014** | The India Newborn Action Plan, developed as a response to the Global Every Newborn Action Plan, targeted for accelerating the reduction of preventable newborn death and stillbirths. The guiding principles for the strategy are Integration, Equity, Gender, Quality of Care, Convergence, Accountability, and Partnerships; built on six pillars of: Pre-conception and antenatal care; Care during labour and child birth; Immediate newborn care; Care of healthy newborn; Care of small and sick newborn; and Care beyond newborn survival (7). |  |
| **Dakshata, 2015** | The *Dakshata* program builds upon the four pause point model of WHO safe childbirth (SCC) checklist, modified for the Indian setting, focuses on improving quality of facility based birthing care by enhancing competency of medical officers, nurses and ANMs by training and mentoring; and ensuring availability of essential commodities and supplies. | A study in Rajasthan explored the effectiveness of WHO SCC and found improvement in adherence to essential practices in the facilities where it was introduced as compared to facilities where it was not. A positive effect on quality of initial assessment and appropriate referral of the women at the admission was also noted (8). |
| **LaQshya, 2017** | The programme focuses on using a quality improvement model for improving care in labour rooms and newborn and is expected to result into delivery of respectful and zero defect care to all pregnant women and newborns, and such improvement is incentivized (9). |  |

1. NHSRC. Programme evaluation of the Janani Suraksha Yojana. New Delhi, India: MOHFW, Government of India; 2011.

2. Ved R, Sundararaman T, Gupta G, Rana G. Program evaluation of the Janani Suraksha Yojna. BMC Proceedings. 2012;6(Suppl 5):O15-O.

3. Singh S, Doyle P, Campbell OM, Oakley L, Rao GR, Murthy G. Interfacility transfer of pregnant women using publicly funded emergency call centre-based ambulance services: a cross-sectional analysis of service logs from five states in India. BMJ Open. 2017;7(6).

4. National Health Mission, Ministry of Health & Family Welfare. Government of India. Janani Shishu Suraksha Karyakaram 2014 [Available from: <http://nhm.gov.in/janani-shishu-suraksha-karyakram.html>.

5. Chaudhary S, Rohilla R, Kumar V, Kumar S. Evaluation of Janani Shishu Suraksha Karyakram scheme and out of pocket expenditure in a rural area of Northern India. Journal of family medicine and primary care. 2017;6(3):477-81.

6. Ministry of Health & Family Welfare. Government of India. Operational Guidelines: Rashtriya Bal Swasthya Karykram 2013 [Available from: <http://nhm.gov.in/images/pdf/programmes/RBSK/Operational_Guidelines/Operational%20Guidelines_RBSK.pdf>.

7. National Health Mission, Ministry of Health & Family Welfare. Government of India. India Newborn Action Plan 2014 [Available from: <http://nhm.gov.in/images/pdf/programmes/inap-final.pdf>.

8. Kumar S, Yadav V, Balasubramaniam S, Jain Y, Joshi CS, Saran K, et al. Effectiveness of the WHO SCC on improving adherence to essential practices during childbirth, in resource constrained settings. BMC pregnancy and childbirth. 2016;16(1):345-.

9. MOHFW. Laqshya: Labour room quality improvement initiative. National Health Mission New Delhi, India: MOHFW; 2017 [Available from: <http://nhm.gov.in/New_Updates_2018/NHM_Components/RMNCH_MH_Guidelines/LaQshya-Guidelines.pdf>
