# Supplementary figures and images for: Care practices and neonatal survival in 52 neonatal intensive care units in Telangana and Andhra Pradesh, India: A cross-sectional study
Source: PLoS Med. 2019 Jul 23;16(7):e1002860. doi: 10.1371/journal.pmed.1002860 (PMC6650044; doi:10.1371/journal.pmed.1002860)

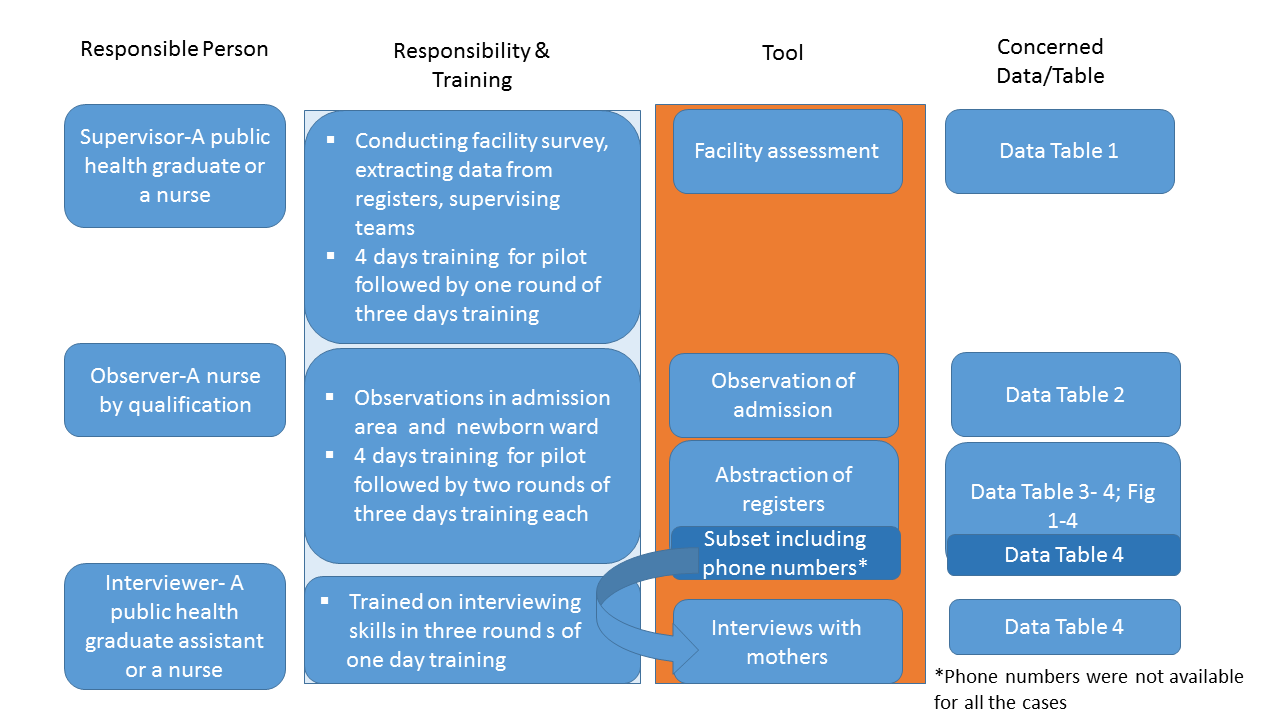

Supplement: S1 Fig — (TIF) [file pmed.1002860.s003.tif]

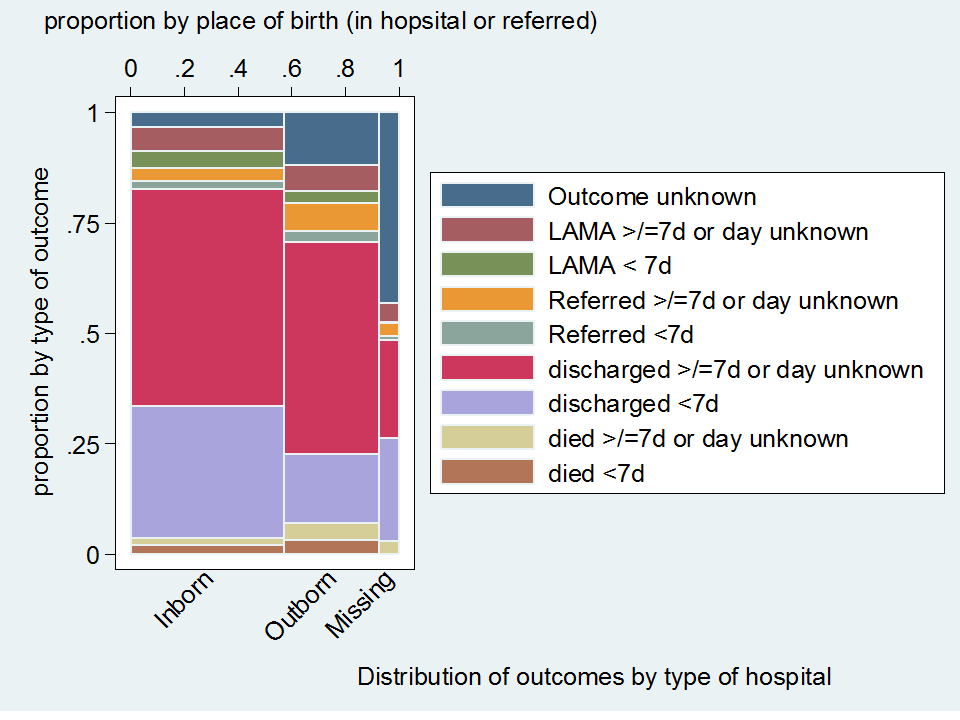

Supplement: S2 Fig — (TIF) [file pmed.1002860.s006.tif]
