# Supplementary material for: Care practices and neonatal survival in 52 neonatal intensive care units in Telangana and Andhra Pradesh, India: A cross-sectional study
Source: PLoS Med. 2019 Jul 23;16(7):e1002860. doi: 10.1371/journal.pmed.1002860 (PMC6650044; doi:10.1371/journal.pmed.1002860)
Supplement: S2 Table — (DOCX) [file pmed.1002860.s004.docx]

S2 Table: Available risk factors and completeness of register observations

| Variable | Categories | % missing |
| --- | --- | --- |
| Inborn/outborn | 57% inborn /36% outborn | 7% missing |
| Cause of admission | 5 causes | 9% missing |
| Gestational age | 20-42 weeks | 57% missing |
| birthweight | 0.5-4.5kg | 41% missing |
| Admission weight | 0.5-5 kg | 48% missing |
