# Supplementary material for: Care practices and neonatal survival in 52 neonatal intensive care units in Telangana and Andhra Pradesh, India: A cross-sectional study
Source: PLoS Med. 2019 Jul 23;16(7):e1002860. doi: 10.1371/journal.pmed.1002860 (PMC6650044; doi:10.1371/journal.pmed.1002860)
Supplement: S3 Table — NICU, neonatal intensive care unit (DOCX) [file pmed.1002860.s005.docx]

S3 Table: Outcomes after admission to neonatal intensive care unit by type of hospital using register data

|  | Public Secondary  N=4,027  (24 hospitals) | | Public Medical Colleges  N=807  (4 hospitals) | | Private Secondary  N=1,314  (14 hospitals) | | Private Medical Colleges  N=672  (5 hospitals) | | Total  N=6,820  (47 hospitals) | | |
| --- | --- | --- | --- | --- | --- | --- | --- | --- | --- | --- | --- |
|  | N | % (95% CI) | N | % (95% CI) | N | % (95% CI) | N |  | N | % (95% CI) |  |
| Died before 7d of life | 80 | 2.0 (1.0-3.9) | 65 | 9.6 (4.4-19.5) | 19 | 1.0 (0.4-2.5) | 0 | 0 | 164 | 3.1 (1.6-5.9) |  |
| Died day 7-28 or day unknown | 77 | 2.3 (0.7-7.2) | 45 | 5.2 (3.3-8.4) | 34 | 3.2 (1.6-6.3) | 12 | 1.5 (0.5-4.9) | 168 | 2.8 (1.4-5.6) |  |
| Discharged before 7d of life | 1200 | 30.7 (20.4-43.4) | 203 | 28.5 (16.4-44.5) | 126 | 9.6 (2.5-30.2) | 132 | 23.7 (5.7-61.4) | 1661 | 27.7 (20.0-37.1) |  |
| Discharged d 7-28 or day unknown | 1865 | 44.9 (33.6-56.7) | 390 | 45.7 (32.9-59.0) | 638 | 44.3 (20.8-70.7) | 289 | 40.4 (19.7-65.2) | 3182 | 44.7 (36.2-53.5) |  |
| Referred before 7d of life | 101 | 2.6 (1.5-4.5) | 16 | 2.0 (1.1-3.6) | 16 | 1.6 (0.5-5.7) | 3 | 0.1 (0-1.1) | 136 | 2.2 (1.4-3.6) |  |
| Referred d7-28 or day unknown | 133 | 3.3 (2.3-4.7) | 19 | 2.1 (1.0-4.3) | 89 | 7.6 (2.6-20.4) | 42 | 5.8 (2.2-14.3) | 283 | 3.7 (2.6-5.2) |  |
| Left against medical advice before 7d of life | 196 | 487 (3.0-7.5) | 14 | 1.8 (0.9-3.4) | 4 | 0.3 (0.1-1.5) | 0 | 0 | 214 | 3.5 (2.2-5.5) |  |
| Left against medical advice d 7-28 or day unknown | 127 | 2.9 (1.6-5.2) | 42 | 4.0 (1.2-13.1) | 65 | 5.3 (2.2-12.6) | 143 | 20.2 (2.9-68.7) | 377 | 4.3 (2.3.8.0) |  |
| Outcome not known | 248 | 6.9 (2.8-14.9) | 13 | 1.2 (0.2-5.6) | 323 | 27.0 (6.9-64.7) | 51 | 8.2 (6.5-10.4) | 635 | 7.9 (3.8-15.7) |  |
