# Supplementary material for: Care practices and neonatal survival in 52 neonatal intensive care units in Telangana and Andhra Pradesh, India: A cross-sectional study
Source: PLoS Med. 2019 Jul 23;16(7):e1002860. doi: 10.1371/journal.pmed.1002860 (PMC6650044; doi:10.1371/journal.pmed.1002860)
Supplement: S4 Table — (DOCX) [file pmed.1002860.s007.docx]

S4_Table: Babies outcome after admission to neonatal care unit assessed by telephonic follow-ups

|  | Baby died | | Baby discharged | | Baby referred | | Left against medical advice | | No outcome reported | Total |
| --- | --- | --- | --- | --- | --- | --- | --- | --- | --- | --- |
|  | < 7days | 7-28 days | < 7days | 7-28 days | < 7days | 7-28 days | < 7days | 7-28 days |  |  |
| Register assessment | 25* | 14* | 194 | 168 | 16 | 7 | 35 | 6 | 516 | 979 |
| Deaths reported at 7 day telephonic follow-up | 24 |  | 4 | 1 | 0 | 0 | 10 | 0 | 20 | 59 |
| Total deaths reported at 28 day telephonic follow-up | 24 | 13 | 6 | 2 | 0 | 1 | 13 | 2 | 41 | 102 |
| 28-day mortality in group |  |  | 2.2% | | 4.3% | | 36.6% | | 8.0% |  |

2 deaths reported in registers were not reported by mothers
