# Supplementary material for: Care practices and neonatal survival in 52 neonatal intensive care units in Telangana and Andhra Pradesh, India: A cross-sectional study
Source: PLoS Med. 2019 Jul 23;16(7):e1002860. doi: 10.1371/journal.pmed.1002860 (PMC6650044; doi:10.1371/journal.pmed.1002860)
Supplement: S1 Data — (DOCX) [file pmed.1002860.s008.docx]

**S 1 Data collection tool LABOUR ROOM FACILITY CHECK LIST**

Before starting your travel to the facility, ensure from your supervisor that the permission for the data collection has been sought from the hospital authorities. Introduce yourself to the Hospital Administrator, explain the purpose of your visit and obtain permission to start your study.

*To be filled by Field Lead:*

| **Variable name in stata** | **Name of Health Care facility** |  |
| --- | --- | --- |
| deleted | **Type of Health Facility** | 1. Sub district hospital  2. District Hospital  3.Government Specialty Hospital  4.Medical College hospital  5.Private Specialty Hospital |
| deleted | **District** |  |
| deleted | **Date of assessment (dd/mm/yyyy)** |  |
| deleted | **Name of Field Lead** |  |

***This check list has to be filled after observing the labour room, asking the responsible MO/ Nurse, by checking the records and by physically verifying the Equipment present in the labour room.***

1. **General Information:**

(*Briefly interview the doctor or staff nurse in-charge and observe for the following)*

| ***Record Number, wherever applicable or Encircle the options given*** | | |
| --- | --- | --- |
| **Variable name in stata** | **Variable Name** | **Options** |
| deleted | Name of the Labour room In-charge person & designation |  |
| deleted | Contact number |  |
| number_beds | No. of beds in labor room: |  |
| del3months | No. of Deliveries in past 3 months (check the records) |  |
| ot | Does the facility has working 24x7 Operation Theatre for Cesarean Section? | YES/NO |
| nbcc | Labour room with New Born Care Corner (NBCC) present? | YES/NO |
| anc | Is the ANC examination room present? | YES/NO |
| receivingarea | Is Receiving area for woman in labour present? | YES/NO |
| predeliveryroom | Is Pre-delivery / Pre-natal room present? | YES/NO |
| postdeliveryroom | Is Post-delivery observation room/Post-natal ward present? | YES/NO |
| labfacilities remarks | Are Supportive Lab facilities present? | YES/NO |
| electricitybackup | Is Electricity / backup 24X7 present? | YES/NO |
| waterdrainage | Is 24X7 Water supply and Drainage present? | YES/NO |
| remarks | Remarks, if any: | |

1. **Human Resources (Count the staff posted in the facility-You may check these details from the roster of the current month) :**

| **S. No** | **Category**  **of staff** | **Number posted in Labour room (whichever row is not applicable write NA)** | | **Numbers trained in** | | | | |
| --- | --- | --- | --- | --- | --- | --- | --- | --- |
|  |  |  |  | Facility Based Newborn Care | Safe child birth checklist | Quality Improvement  (Institute for Healthcare Improvement or any other certificate) | Skilled Birth Attendant | Any other trainings *(please specify)* |
|  | No. of Gynaecologists/ Obstetricians | Day Shift  *obsday* |  | *obsdayfbnc* | *obsdayscbc* | *obsdayqi* | *obsdaysba* | *obsdayothers* |
|  |  | Night Shift  *obsnight* |  | *obsnightfbnc* | *obsnightscbc* | *obsnightqi* | *obsnightsba* | *Obsnightothers* |
| 1.1 | *On call Gynaecologists/ Obstetricians 24x7* | Day Shift  *oncallobsday* |  | *oncallobsdayfbnc* | *oncallobsdayscbc* | *oncallobsdayqi* | *oncallobsdaysba* | *oncallobsdayothers* |
|  |  | Night Shift  *oncallobsnight* |  | *oncallobsnightfbnc* | *oncallobsnightscbc* | *oncallobsnightqi* | *oncallobsnightsba* | *oncallobsnightothers* |
|  | Medical Officers (MBBS) | Day Shift  *moday* |  | *modayfbnc* | *modayscbc* | *modayqi* | *modaysba* | *modayothers* |
|  |  | Night Shift  *monight* |  | *monightfbnc* | *monightscbc* | *monightqi* | *monightsba* | *monightothers* |
|  | On call Anaesthesiologist 24x7 in obstetric OT | Day Shift  *anaesday* |  | *anaesdayfbnc* | *anaesdayscbc* | *anaesdaysba* | *anaesdayqi* | *anaesdayothers* |
|  |  | Night Shift  *anaesnight* |  | *anaesnightfbnc* | *anaesnightscbc* | *anaesnightqi* | naesnightsba | *anaesnightothers* |
|  | On call Paediatrician 24x7 in Labour room/ obstetric OT | Day Shift  *paedday* |  | *paeddayfbnc* | *paeddayscbc* | *paeddayqi* | *paeddaysba* | *paeddayothers* |
|  |  | Night Shift  *paednight* |  | *paedsnightfbnc* | *paednightscbc* | *paednightqi* | *paednightsba* | *paednightothers* |
|  | Nurses posted in Labor room | Day Shift  *nurseday* |  | *nursedayfbnc* | *nursedayscbc* | *nursedayqi* | *nursedaysba* | *nursedayothers* |
|  |  | Night Shift  *nursenight* |  | *nursenightfbnc* | *nursenightscbc* | *nursenightqi* | *nursenightsba* | *nursenightothers* |
| 5.1 | *Pediatric Nurse posted in LR* | Day Shift  *paednurseday* |  | paednursedayfbnc | paednursedayscbc | paednursedayqi | paednursedaysba | paednursedayothers |
|  |  | Night Shift  *paednursenight* |  | *paednursenightfbnc* | *paednursenightscbc* | *paednursenightsba* | *paednursenightqi* | *paednursenightothers* |
|  | Delivery assistants | Day Shift  *deliveryasstday* |  | - | - | - | - | - |
|  |  | Night Shift  *deliveryasstnight* |  | - | - | - | - | - |
|  | Cleaners | Day Shift  *cleanersday* |  | - | - | - | - | - |
|  |  | Night Shift  *cleanersnight* |  | - | - | - | - | - |

1. **List of Protocols:** *verify all the protocols physically and note down in the remarks column that the protocol is displayed in the maternity ward*

| **S. No** | **List of Protocols** | Present   1. Yes 2. No | 1. Hospital Specified 2. Adopted from Standards | 1.Common Understanding  2. Written Guideline | Displayed   1. Yes 2. No |
| --- | --- | --- | --- | --- | --- |
|  |  |  |  |  |  |
|  | Display of SBA quality protocols (physically verify)   1. Hand washing 2. Infection Prevention 3. Partograph 4. Active Management of Third Stage of labor 5. Kangaroo Care 6. Blood transfusion 7. Pre-term labor 8. Ante natal steroids | handwash_protocol  infecprev_protocol  parto_protocol amtsl_protocol  kmc_protocol bt_protocol  preterm_protocol  steroids_protocol | handwash_type  infecprev_type  parto_type  amtsl_type  kmc_type  bt_type  preterm_type  steroids_type | handwash_dissemination  infecprev_dissemination  parto_dissemination  amtsl_dissemination  kmc_dissemination  bt_dissemination  preterm_dissemination  steroids_dissemination | handwash_display  infecprev_display  parto_display  amtsl_display  kmc_display  bt_display  preterm_display  steroids_display |
|  | Record if any other protocols are displayed   1. *anyother_protocol1* 2. *anyother_protocol2* 3. *anyother_protocol3* 4. *anyother_protocol4* | | | | |

1. **Cleanliness and Infection Control**

| **S. No** | **Criteria** | **Present**  **YES/NO** | **Remarks if any**  ***** Observe and write down only the items which are absent |
| --- | --- | --- | --- |
|  | Verify the cleanliness and absence of dust, blood, trash on: floors, walls, top of doors, tables, foot rest, lamps, tabletops, lights, examination and delivery table, counters, non-critical equipment, baby resuscitation equipment  *clean* | YES/NO | *items_notclean* |
| 1.1 | Are the services for cleaning contracted out?  *clean_contract* | YES/NO | *clean_contract_remarks* |
| 1.2 | Is there any protocol for cleanliness?  *clean_protocol* | YES/NO | *clean_protocol_remarks* |
|  | Dirt room (room for keeping soiled linens, bedpans before sending them for sterilization)  *Dirtroom* | YES/NO | *dirtroom_remarks* |
|  | Clean Utility room  (area demarcated for keeping up sterile supplies received after sterilization)  *Utilityroom* | YES/NO | *utilityroom_remarks* |
|  | Attached hand washing area with elbow operated taps in Labour room  *elbowtap* | YES/NO | *elbowtap_remarks* |
|  | Hand-washing area for the admitted clients in Labour room  *handwashclients* | YES/NO | *handwash_remarks* |
|  | Drinking water for patients  *Drinkwater* | YES/NO | *drinkwater_remarks* |
|  | Clean usable Toilets for patients in Labour room  *toiletpatient* | YES/NO | *toiletpatient_remarks* |
|  | Clean usable Toilets for Staff in Labour room  *Toiletstaff* | YES/NO | *toiletstaff_remarks* |
|  | Check for presence of Hand washing supplies: Soap, Clean towels Sterilized, alcohol based hand wash  *handwashsupply* | YES/NO | *handwashsupply_remarks* |
|  | Instrument sterilizer, Jar for forceps  *sterilizerjar* | YES/NO | *sterilizerjar_remarks* |

1. **General Equipment list for Labour Room:**

| **S. No** | **Equipment’s** | **If Present**  **YES/NO** | ***** Observe and write down only the items which are absent |
| --- | --- | --- | --- |
| **Basic equipment** | | | |
|  | Sphygmomanometer (BP apparatus)  *Sphygmo* | YES/NO | *sphygmo_remarks* |
|  | Stethoscope  *Sthetho* | YES/NO | *sthetho_remarks* |
|  | Foeto-scope/Foetal Doppler  *Foetoscope* | YES/NO | *foetoscope_remarks* |
|  | Adult Thermometer  *Thermometer* | YES/NO | *thermometer_remarks* |
|  | Newborn Thermometer  *newbornthermo* | YES/NO | *newbornthermo_remarks* |
|  | Sterilizer Equipment  *Sterilizer* | YES/NO | *sterilizer_remarks* |
|  | Consumables Like: |  |  |
|  | Gloves  *Gloves* | YES/NO | *gloves_remarks* |
|  | Apron  *Apron* | YES/NO | *apron_remarks* |
|  | Disposable sterilized gowns  *Gowns* | YES/NO | *gowns_remarks* |
|  | Cotton  *cotton* | YES/NO | cotton_remarks |
|  | Gauze  *Gauze* | YES/NO | *gauze_remarks* |
|  | Sanitary Napkins  *Napkins* | YES/NO | *napkins_remarks* |
|  | Catgut  *Catgut* | YES/NO | *catgut_remarks* |
|  | IV Drip Sets  *ivdrip* | YES/NO | *ivdrip_remarks* |
|  | Needle  *Needle* | YES/NO | *needle_remarks* |
|  | Cord Clamp  *cordclamp* | YES/NO | *cordclamp_remarks* |
|  | Leucoplast tape  *Leucoplast* | YES/NO | *leucoplast_remarks* |
|  | Suction machine (handheld or electric)  suction | YES/NO | *suction_remarks* |
|  | Newborn weighing machine/scale  *Weighing* | YES/NO | *weighing_remarks* |
|  | Pulse oximeter  *Oximeter* | YES/NO | *oximeter_remarks* |
|  | Lamp – wall mounted or side  *Lamp* | YES/NO | *lamp_remarks* |
|  | Refrigerator  refrigerator | YES/NO | refrigerator_remarks |
| **Absolute minimum equipment for delivery** | | | |
|  | Autoclaved delivery set for each delivery  *Deliveryset* | YES/NO | *deliveryset_remarks* |
|  | Labour table  *Labourtable* | YES/NO | *labourtable_remarks* |
|  | Mattress  *mattress* | YES/NO | *mattress_remarks* |
|  | Sheet  *Sheet* | YES/NO | *sheet_remarks* |
|  | Pillow (numbers as per case load)  *Pillow* | YES/NO | *pillow_remarks* |
|  | Macintosh  *macintosh* | YES/NO | *macintosh_remarks* |
|  | Foot-rest  *Footrest* | YES/NO | *footrest_remarks* |
|  | Kelly’s pad  *kellypad* | YES/NO | *kellypad_remarks* |
|  | IV drip stand | YES/NO |  |
|  | Equipment for adult resuscitation  *resuscitation* | YES/NO | *resuscitation_remarks* |
|  | Oxygen cylinder and concentrator  *Oxygen* | YES/NO | *oxygen_remarks* |
| **Absolute minimum for care of Neonate** | | | |
|  | Cloth or Towel to dry baby  *toweltodry* | YES/NO | *toweltodry_remarks* |
|  | Blanket/towel to wrap baby  *Toweltowrap* | YES/NO | *toweltowrap_remarks* |
|  | Radiant Warmer  *radiantwarmer* | YES/NO | *radiantwarmer_remarks* |
|  | Equipment for neonatal resuscitation |  |  |
|  | Bag and mask  *Bagmask* | YES/NO | *bagmask_remarks* |
|  | Mucus aspirator  *mucusaspirator* | YES/NO | *mucusaspirator_remarks* |
|  | Pediatric laryngoscope  *laryngoscope* | YES/NO | *laryngoscope_remarks* |
|  | Wheel chair  *Wheelchair* | YES/NO | *wheelchair_remarks* |
|  | Patient's trolley  *Trolley* |  | *trolley_remarks* |
|  | Wall clock  clock ivdripsets ivdripsets_remarks gloves1 | YES/NO | *clock_remarks* |
|  | In the opinion of Medical Officer/Staff Nurse, Is the infrastructure and logistics available is sufficient as per the caseload?  *Opinionmo* | YES/NO | *opinionmo_remarks* |

1. **Equipment for Delivery detail:**

Observe and tick the items which are present *(If tray is not ready at the time of visit, ask the nurse in-charge about the items that are to be kept in the tray)*

| S. No | **Particulars of trays** | | | | |
| --- | --- | --- | --- | --- | --- |
|  | **Sterilized Delivery tray** | | | | |
|  | Gloves,  Scissor,  Artery forceps,  Cord clamp,  Sponge holding forceps,  Urinary catheter  *gloves1*  *scissor*  *artery*  *cord*  *sponge*  urinary | YES/NO  YES/NO  YES/NO  YES/NO  YES/NO  YES/NO | Bowl for antiseptic lotion,  Gauze pieces and cotton swabs,  Speculum,  Sanitary pads,  Kidney tray,  *Bowl*  *gauze1*  *speculum*  *sanitary*  *kidney* | | YES/NO  YES/NO  YES/NO  YES/NO  YES/NO |
|  | **Sterilized Episiotomy tray** | | | | |
|  | Inj. Xylocaine 2%,  10 ml disposable syringe with needle,  Episiotomy scissor,  Kidney tray,  Artery forceps  Chromic catgut no. 0.  *xylocaine*  *syringeneedle*  *episcissor*  *kidneytray*  *arteryforceps*  *chromiccatgut* | YES/NO  YES/NO  YES/NO  YES/NO  YES/NO  YES/NO | Allis forceps,  Sponge holding forceps,  Toothed forceps,  Needle holder,  Needle (round body and cutting),  *allisforceps*  *spongeholder*  *toothedforceps*  *needleholder*  *needle1* | YES/NO  YES/NO  YES/NO  YES/NO  YES/NO | |
|  | **Medicine tray** | | | | |
|  | Inj. Oxytocin (to be kept in fridge)  Cap Ampicillin 500 mg  Tab Metronidazole 400 mg  Tab Paracetamol  Tab Ibuprofen  Tab B complex  IV fluids  Tab. Misoprostol 200 micrograms  *oxytocin*  *capampicillin*  *tabmetro*  *paracetamol*  *ibuprofen*  *bcomplex*  *ivfluids*  *tabmisoprostol* | YES/NO  YES/NO  YES/NO  YES/NO  YES/NO  YES/NO  YES/NO  YES/NO | Inj. Gentamycin  Vit K,  Inj. Betamethason  Ringer lactate  Normal Saline  Inj. Hydrazaline  Tab. Nefidepin  Tab. Methyldopa.  *gentamicin*  *vitk*  *betamethason*  *lactate*  *saline*  *hydrazaline*  *nefidepin*  *methyldopa* | YES/NO  YES/NO  YES/NO  YES/NO  YES/NO  YES/NO  YES/NO  YES/NO | |
|  | **Emergency medicine** | | | | |
|  | Inj. Magsulfate 50%,  Inj. Calcium gluconate-10%,  Inj. Dexamethasone,  Inj. Ampicillin,  Inj. Gentamicin,  Inj. Metronidazole,  Inj. Lignocaine-2%,  *magsulfate*  *calcium*  *dexamethasone*  *ampicillin*  *gentamycin*  *metronidazole*  *lignocaine* | YES/NO  YES/NO  YES/NO  YES/NO  YES/NO  YES/NO  YES/NO | Inj. Adrenaline,  Inj. Hydrocortisone Succinate ,  Inj. Diazepam,  Inj. Pheneramine maleate ,  Inj. Phenergan,  Ringer lactate,  normal saline.  *adrenaline*  *hydrocortisone*  *diazepam*  *pheneramine*  *phenergan*  *rlactate*  *normalsaline* | YES/NO  YES/NO  YES/NO  YES/NO  YES/NO  YES/NO  YES/NO | |
|  | Does patient have to pay or have to sign for drugs?  *If yes, specify the name of drug*  *pay_signdrugs* | YES/NO | Is there any medicine that had been kept locked in shelves?  *Specify*  *pay_signspecify* | YES/NO | |
|  | **Miscellaneous** | | | | |
|  | IV sets with 16-gauge needle at least two,  Controlled suction catheter  *ivset*  *suctioncatheter* | YES/NO  YES/NO | Mouth gag,  IV Canula  *mouthgag*  *ivcanula* | YES/NO  YES/NO | |

1. **Infection prevention:**

|  | Demarcated area for keeping slippers for the hospital staff and relatives and slippers to be used for entering the labour/pre-labour room  *Areaslippers* | Yes/ No/Yes but not in use |
| --- | --- | --- |
|  | Are they practicing correct method of Bio medical waste management  *Bmw* | Yes/ No/Yes but not in use |
|  | Syringe cutter present  *syringecutter* | Yes/ No/Yes but not in use |
|  | 1% bleaching powder solution  *Bleaching* | Yes/ No/Yes but not in use |
|  | Bio medical waste bins (Black, Blue and Red/Yellow) and bags present?  *Wastebins* | Yes/ No/Yes but not in use |
|  | Frequency of disposal (in days)  Disposaldays |  |
|  | Puncture Proof Container present  puncture_container | Yes/ No/Yes but not in use |
|  | Personal protective covers for waste handlers present?  protectivecovers | Yes/ No/Yes but not in use |
|  | Sterile gown given to patient going for delivery?  Sterilegown | Yes/ No/Yes but not in use |

1. **List of Records:**

| **S. No** | **Record** | **Available**  **Yes/No** | **Updated**  **Yes/No** | **Completely Filled**  **Yes/No** |
| --- | --- | --- | --- | --- |
|  | Partograph  *parto*  otregister | Yes/No | *parto_update* | *parto_complete* |
|  | Case sheets  *casesheet* | Yes/No | *casesheet_update* | *casesheet_complete* |
|  | Labor Room Record Register  *lrrecord* | Yes/No | *lrrecord_update* | *lrrecord_complete* |
|  | Operation Theatre Register  *otregister* | Yes/No | *otregister_update* | *otregister__complete* |
|  | Newborn Register | Yes/No | *newbornregister_update* | newbornregister__complete |
|  | Maternal Death Register  *mdregister* | Yes/No | *mdregister_update* | *mdregister__complete* |
|  | Pregnant women Refer in/ | Yes/No | *pregreferin_update* | *pregreferin__complete* |
|  | Pregnant women Refer out  *pregreferoutregister* | Yes/No | *pregreferout_update* | *pregreferout__complete* |
|  | Newborn Refer in  *nbreferinregister* | Yes/No | *nbreferin_update* | *nbreferin__complete* |
|  | Newborn Refer out  *nbreferoutregister* | Yes/No | *nbreferout_update* | *nbreferout__complete* |

1. **Rough Sketch of Labor Room:**
