# Supplementary material for: Care practices and neonatal survival in 52 neonatal intensive care units in Telangana and Andhra Pradesh, India: A cross-sectional study
Source: PLoS Med. 2019 Jul 23;16(7):e1002860. doi: 10.1371/journal.pmed.1002860 (PMC6650044; doi:10.1371/journal.pmed.1002860)
Supplement: S2 Data — NICU, neonatal intensive care unit (DOCX) [file pmed.1002860.s009.docx]

**S 2 Data collection tool Facility Survey of SNCU (Sick Newborn Care Unit)/**

**NICU (Neonatal Intensive Care Unit)**

Before starting your study at the respective facility, ensure from your supervisor that the permission for the data collection has been sought from the hospital authorities. Introduce yourself to the Hospital Administrator, explain the purpose of your visit and obtain permission to start your study.

*To be filled by Field Lead:*

| **Variable name in stata** | **Variable** | **Response** |
| --- | --- | --- |
| *deleted* | **Name of Health Care facility:** |  |
| *Deleted* | **Date of assessment (dd/mm/yyyy):** |  |
| *Deleted* | **Name of Field Lead:** |  |

1. **Briefly interview the doctor or staff nurse in-charge for the following.**

| **Variable name in stata** | **Variable** | **Response** |
| --- | --- | --- |
| *deleted* | Name of Paediatric Facility in-charge: |  |
| *Deleted* | Contact number |  |
| *Deleted* | Name of SNCU in-charge & Designation: |  |
| *deleted* | Contact number |  |
| *sncu_year* | Year in which SNCU/NICU started |  |
| *beds_count* | Number of beds in the SNCU |  |
| *admissions_count* | Number of admissions in the SNCU in past 3 months |  |

1. **Human resources for SNCU (Count the staff posted in the facility-You may check these details from the roster of the current month)**

| **Variable name in stata** | **Variable** | | | | |
| --- | --- | --- | --- | --- | --- |
|  | Category of staff | Mention actual no of posted at the facility (which ever row is not applicable write NA) | Number present during the visit | | |
|  |  |  | Day shift | Noon shift | Night shift |
| *regularpostedpaed* | Number of Paediatricians posted full time in the SNCU |  |  |  |  |
| *oncallpaed* | Number of Paediatricians on-call |  |  |  |  |
| *neonatal* | Number of neonatologist available full time or on call |  |  |  |  |
| *paednurse* | Number of Paediatric / neonatal staff nurses posted full time in the SNCU |  |  |  |  |
| *oncallpaednurse* | Number of Paediatric / neonatal staff nurses on-call |  |  |  |  |
| *paed_qi* | Number of Paediatricians of the total, trained for QI |  |  |  |  |
| *nurse_qi* | Number of staff nurses of the total, trained for QI |  |  |  |  |

1. **Clinical Support services:**

| **Variable name in stata** | **Variable** | **Available** |
| --- | --- | --- |
| *housekeeping* | Housekeeping services | N  Y |
| *ambulance* | Ambulance services | N  Y |
| *cssd* | Autoclaving / CSSD | N  Y |
| *bmw* | Management of Bio-Medical Waste (BMW) | N  Y |
| *pharma* | Pharmacy | N  Y |
| *referral referralcenter* | Referral services  (if yes, mention the name of the most commonly, referred to centre) ___________________ | N  Y |

**Ask the doctor or staff nurse in-charge if he/she can help you look around the SNCU/NICU. Record your observations using this checklist and write comments wherever required.**

1. **Functional area**

| **Variable name in stata** | **Area** | **Available** | **Comments/**  **Variable name in stata** |
| --- | --- | --- | --- |
| *receiving* | Baby receiving room | Y  N | *receivingremarks* |
| *breastfeeding* | Breast feeding area | Y  N | *breastfeedingremarks* |
| *invasive* | Special room for invasive procedures | Y  N | *invasiveremarks* |
| *isolation* | Isolation room (for infected babies) | Y  N | *isolationremarks* |
| *ivfluidpreparationroom* | I/V fluid preparation room | Y  N | *ivfluidpreparationroomremarks* |
| *kmc* | Room for Kangaroo care | Y  N | *kmcremarks* |
| *stablearea* | Room for non-critical/stable babies to stay with their mothers before discharge  (step down room) | Y  N | *stablearearemarks* |

1. **Equipment (physically observation and ask to the duty staff nurse, if needed)**

| **Variable name in stata** |  | | **Availability** | **If available, how many?/**  **Variable name in stata** | **Comments/**  **Variable name in stata** |
| --- | --- | --- | --- | --- | --- |
| *stethoscope* | **Monitoring Equipments** | Stethoscope with neonatal chest piece | N  Y | *stethescopeno* | *stethescoperemarks* |
| *bpmonitors* |  | Non-invasive BP monitors | N  Y | *bpmonitorsno* | *bpmonitorsremarks* |
| *thermo* |  | Low reading clinical thermometers | N  Y | *thermono* | *thermoremarks* |
| *roomthermo* |  | Room thermometers | N  Y | *roomthermono* | *roomthermoremarks* |
| *electronic_scale* |  | Electronic weighing scale | N  Y | *electronic_scaleno* | *electronic_scaleremarks* |
| *mechanical_scale* |  | Mechanical weighing scale | N  Y | *mechanical_scaleno* | *mechanical_scaleremarks* |
| *ventilators1* | **Equipments used for managing** | Ventilators | N  Y | *ventilatorsno1* | *ventilatorsno1remarks* |
| *radiant_warmer* |  | Radiant warmer (with Servo control) | N  Y | *radiant_warmerno* | *radiant_warmerremarks* |
| *phototherapy* |  | Phototherapy unit | N  Y | *phototherapyno* | *phototherapyremarks* |
| *cpap* |  | CPAP and short term ventilation | N  Y | *cpapno* | *cpap_remarks* |
| *self_inflating* | **Resuscitation equipments** | Self-inflating bag | N  Y | *self_inflatingno* | *self_inflatingremarks* |
| *mucus_trap* |  | Foot operated suction apparatus/mucus trap | N  Y | *mucus_trapno* | *mucus_trapremarks* |
| *oxygen_cylinder* |  | oxygen cylinders/Central supply | N  Y | *oxygen_cylinderno* | *oxygen_cylinderremarks* |
| *generators* | **General equipments** | Generators | N  Y | *generatorsno* | *generatorsremarks* |
| *ups* |  | UPS | N  Y | *upsno* | *upsremarks* |
| *refrigerator* |  | Refrigerator | N  Y | *refrigeratorno* | *refrigeratorremarks* |
| *wall_clock* |  | Wall clock with second’s hand | N  Y | *wall_clockno* | *wall_clockremarks* |
| *auto_claving_equipment* |  | Autoclaving equipments | N  Y | *auto_claving_equipmentno* | *auto_claving_equipmentremarks* |
| *surgical_instruments* |  | Surgical instruments | N  Y | *surgical_instrumentsno* | *surgical_instrumentsremarks* |
| *syringe_hub* |  | Syringe hub cutters | N  Y | *syringe_hubno* | *syringe_hubremarks* |
| *ecg_machine* |  | ECG machine | N  Y | *ecg_machineno* | *ecg_machineremarks* |
| *air_conditioners* |  | Air conditioners | N  Y | *air_conditionersno* | *air_conditionersremarks* |

1. **Drug listing: To be checked physically by seeking help from Duty staff nurse.**

| **Variable name in stata** | **Emergency drugs** | **Availability** | **Remarks/**  **Variable name in stata** |
| --- | --- | --- | --- |
| *injantibio* | Antibiotics-Inj | N  Y | *injantibioremarks* |
| *ampicilin* | Ampicillin with Cloxacillin | N  Y | *ampiremarks* |
| *injampi* | Inj. Ampicillin | N  Y | *injampiremarks* |
| *injcefato* | Inj. Cefotaxime | N  Y | *injcefatoremarks* |
| *injgenta* | Inj. Gentamycin | N  Y | *injgentaremarks* |
| *amoxy* | Amoxycillin-Clavulanic Suspension | N  Y | *amoxyremarks* |
| *analgesics* | Analgesics & antipyretics-Paracetamol | N  Y | *analgesicremarks* |
| *ivfluids1* | IV Fluids-5%, 10%, 25%Dextrose | N  Y | *ivfluidsremarks1* |
| *saline* | Normal saline | N  Y | *salineremarks* |
| *electrolyte* | Drugs for electrolyte imbalance-Inj | N  Y | *electrolyteremarks* |
| *potassium* | Potassium Chloride 15% | N  Y | *potassiumremarks* |
| *gluconate* | Inj. Calcium Gluconate 10% | N  Y | *gluconateremarks* |
| *sulphonate* | Inj. Magnesium Sulphate 50% | N  Y | *sulphonateremarks* |
| *adneralinestatus* | Adrenaline(1:10000) | N  Y | *adneralineremarks* |
| *naloxone* | Inj. Naloxone | N  Y | *naloxoneremarks* |
| *bicarbonate* | Sodium Bicarbonate | N  Y | *bicarbonateremarks* |
| *amnophyline* | Injection Aminophy lline | N  Y | *amnophylineremarks* |
| *phinobarbitone* | Phenobarbitone (Injection+oral) | N  Y | *phinobarbitoneremarks* |
| *dexamethosone* | Inj. Dexamethasone | N  Y | *dexamethosoneremarks* |
| *phenytoin* | Inj. Phenytoin | N  Y | *phenytoinremarks* |
| *new_drug* | other newborn drugs-Vitamin K | N  Y | *new_drugremarks* |
| *hydrocortisone* | Injection Hydrocortisone | N  Y | *hydrocortisoneremarks* |

1. **Records to be verified at SNCU/NICU:**

| **Variable name in stata** | **Name of the record** | **Available (tick)** | **Updated**  **(tick)** | **Completed**  **(tick)** | **Comments** |
| --- | --- | --- | --- | --- | --- |
| *admin*  *adminupdate admincomplete adminremarks* | **Admission register** | N  Y | N  Y | N  Y |  |
| *nbdeath nbdeathupdate nbdeathcomplete nbdeathremarks* | **Newborn Death Register** | N  Y | N  Y | N  Y |  |
| *refer*  *referupdate refercomplete referremarks* | **Refer in / out Register** | N  Y | N  Y | N  Y |  |
| *otherspecify*  *otherspecify_1*  *otherspecifyupdate otherspecifycomplete otherspecifyremarks* | **If other, Specify** |  |  |  |  |

1. **Rough Sketch of SNCU:**
