# Supplementary material for: Care practices and neonatal survival in 52 neonatal intensive care units in Telangana and Andhra Pradesh, India: A cross-sectional study
Source: PLoS Med. 2019 Jul 23;16(7):e1002860. doi: 10.1371/journal.pmed.1002860 (PMC6650044; doi:10.1371/journal.pmed.1002860)
Supplement: S3 Data — (DOCX) [file pmed.1002860.s010.docx]

**S3 Data Collection Tool Observation Checklist for SNCU**

| **Read carefully before starting your day of observation**   1. You should spend at least 4 dedicated hours daily for observation in SNCU/ NICU ward room. 2. Inform and explain about your visit to person i/c for the day and show the permission letter. 3. Take the consent of all the health staff on duty at the time of observation before you begin your observations. 4. Find a place where you can sit/stand comfortably and observe the activities of staff present in SNCU/ NICU ward. Whenever a new case arrives in to the SNCU/ NICU, you should go and observe the activities in the SNCU/ NICU receiving area. 5. Don’t disturb the activities of the staff even if you miss on any observation 6. Don’t intervene in between even if you feel that the activity is not performed as per the standard 7. Observe and note down the observations 8. In case you missed the name of the administered drug or any such information given, you may confirm it later with the person I/c or note it down from the case sheet 9. You may extend your observation time if any new Baby arrives in last 15 minutes of the observation hours. Try to observe complete admission/delivery process for the Baby 10. There are two categories of observations that you have to make: 11. Observations if any health staff visits the baby in SNCU 12. Observations if mother/relative visits the baby in SNCU 13. Observation for new admission |
| --- |

| **Variable in stata** |  |  |
| --- | --- | --- |
| *deleted* | **Name of Health Care facility** |  |
| *deleted* | **Date of assessment (dd/mm/yyyy)** |  |
| *deleted* | **Name of Observer** |  |

**SNCU/NICU RECEIVING AREA**

1. **Observations for a new arrival in the receiving area of SNCU/NICU**

| **Variable in stata** | **Si. No. for the period of observation** |  | |
| --- | --- | --- | --- |
| *deleted* | Date and time of arrival |  | |
| *deleted* | *Baby SNCU/NICU admission id.* |  | |
| *deleted* | *Baby name.* |  | |
| *deleted* | *Mothers name* |  | |
| *If you miss baby’s identifiers, record these after the admission process is complete and baby is stabilized. Do not intervene in between.* | | | |
| *condition* | What is the general condition of the baby? | 1. Awake / Crying 2. Unconscious | |
| *accompaniedby* | Who is accompanying? | 1. Only relative 2. Health personnel 3. Ambulance personnel | |
| *observedby* | Who is observing the baby?  (multiple choice possible) | 1. Medical officer 2. Paediatrician 3. Nurse 4. Other attendant | |
| *attendno* | How many health personnel are attending the baby? |  | |
| *attendpaed* | Pediatrican is attending the baby | (tick) | (system time) |
| *Carefully observe what is being performed to the baby and check if any of the below is being done.* | | | |
| *observecondition* | General condition of the baby is being observed | (tick) | (system time) |
| *historytaken* | Examiner is eliciting the history | (tick) | (system time) |
| *birthdateasked* | Birth date of baby asked or looked for in records | (tick) | (system time) |
| *weighttaken* | Birth weight of baby asked or looked for in records | (tick) | (system time) |
| *motherbaby_compbirth* | Complication in mother or baby at the time of birth, asked or looked for in records | (tick) | (system time) |
| *hygienebefore* | Hand hygiene before | (tick) | (system time) |
| *auscultation* | Auscultation of the baby being done | (tick) | (system time) |
| *temptaken* | Temperature of the baby being taken | (tick) | (system time)  **Record time** |
| *birthweightasked* | Weight of the baby being taken | (tick) | (system time) |
| *ivdone* | I/v insertion being done | (tick) | (system time)  **Record time** |
| *resuscdone* | Any resuscitation provided | (tick) | (system time) |
| *treatmentgiven* | Any treatment provided | (tick) | |
| *babywarm* | Baby kept warm during above process | (tick) | |
| *sentbackrefer* | Baby sent back or referred | (tick) | (system time) |
| *paperwork* | Admission paper work being done | (tick) | |
| *babyshiftnicu* | Baby shifted in SNCU/ NICU | (tick) | (system time)  **Record time** |
| *cpap* | Baby put on CPAP | (tick) | (system time) |
| *adjusttempwarmer* | Warmer temperature adjusted | (tick) | |
| *hygieneafter* | Handhygiene after | Y/N | |
