# Supplementary material for: Care practices and neonatal survival in 52 neonatal intensive care units in Telangana and Andhra Pradesh, India: A cross-sectional study
Source: PLoS Med. 2019 Jul 23;16(7):e1002860. doi: 10.1371/journal.pmed.1002860 (PMC6650044; doi:10.1371/journal.pmed.1002860)
Supplement: S4 Data — (DOCX) [file pmed.1002860.s011.docx]

**S 11 Abstraction of NICU registers**

| **1. Ask the Sister/Nurse in-charge to share with you the SNCU record register/SNCU data entry software**  **2. Line-list all the admissions in SNCU/NICU for past one month with the mentioned details.** |
| --- |

| **Name of Health Care facility:** |  |
| --- | --- |
| **Date of assessment (dd/mm/yyyy):** |  |
| **Name of Observer** |  |

**Line listing of all the cases admitted in SNCU/NICU in past one month including new admissions at the time of one month observation. Information to be extracted from admission register, referral register and death registers in SNCU/NICU** (**To be filled by Observer)**

| **Variable name in data base** |  | **Patient X** | **Patient Y** | **Patient Z** |
| --- | --- | --- | --- | --- |
| SNo | **Admission ID** |  |  |  |
| statecode | **Telangana / Andhra** |  |  |  |
| hcode | **Hospital number** |  |  |  |
| District | **District name** |  |  |  |
| Type | **Hospital Type** |  |  |  |
| Type_sncu | **NICU type** |  |  |  |
| admissiondate | **Date and time of admission in SNCU/NICU** |  |  |  |
| deleted | **Name of baby** |  |  |  |
| Not captured | **Gender of baby** |  |  |  |
| Not captured | **Name of mother of the baby** |  |  |  |
| deleted | **Contact number 1** |  |  |  |
| deleted | **Contact number 2** |  |  |  |
| birthdate | **Date and time of birth** |  |  |  |
| Not captured | **Hours of birth** |  |  |  |
| Not captured | **Gestational age in weeks** |  |  |  |
| Not captured | **Birth weight** |  |  |  |
| In_outborn | **Inborn/ Out born** |  |  |  |
|  | **Diagnosis at admission in NICU/SNCU (TICK IF YES) , *multiple choice possible*** |  |  |  |
| diag_lbw | 1. **LBW** |  |  |  |
| diag_preterm | 1. **Preterm** |  |  |  |
| diag_asphyxia | 1. **Birth asphyxia** |  |  |  |
| diag_sepsis | 1. **Sepsis** |  |  |  |
| diag_nnj | 1. **Neonatal jaundice** |  |  |  |
| diag_malformation | 1. **Malformation** |  |  |  |
| diag_others | 1. **If Others, Specify** |  |  |  |
|  | **Maturity** |  |  |  |
| Not captured | 1. **Pre-Term(<37 wks)** |  |  |  |
| Not captured | 1. **Full Term (37-42 wks)** |  |  |  |
| Not captured | 1. **Post Term(=> 42 wks)** |  |  |  |
| Not captured | **Time of first temperature measurement in NICU/SNCU** |  |  |  |
| Not captured | **Weight at time of admission in NICU/SNCU** |  |  |  |
| outcome | **Outcome- i) admitted, ii) discharged,**  **iii) referred, iv) dead, v) LAMA** |  |  |  |
| outcomedate | **Date of outcome** |  |  |  |
| Not captured | **If outcome was death,**  **What was the cause of death?**  **Cause of death 1** |  |  |  |
| Not captured | **Cause of death 2** |  |  |  |
| Not captured | **Cause of death 3** |  |  |  |
