# Supplementary material for: Care practices and neonatal survival in 52 neonatal intensive care units in Telangana and Andhra Pradesh, India: A cross-sectional study
Source: PLoS Med. 2019 Jul 23;16(7):e1002860. doi: 10.1371/journal.pmed.1002860 (PMC6650044; doi:10.1371/journal.pmed.1002860)
Supplement: S5 Data — (DOCX) [file pmed.1002860.s012.docx]

**S 5 Data Collection Tool Structured telephone interview guideline**

*(To interview mother/immediate care-taker with live birth recruited from Post-natal ward or mother/immediate care-taker of babies discharged from SNCU)*

|  | **7 day** | **28 day** |
| --- | --- | --- |
| 1. Name of the interviewer: 2. Interview date: 3. Interview start time: 4. Interview end time: |  |  |

| **S. No.** | **Question** | **Record answer in the space/options given below** |
| --- | --- | --- |
| 1. **Patient Details** | | |
| **A.1** | **Patient ID**  *(Enter the patient ID- This will link the previously gathered information of the patient)* |  |
| **deleted** | **Name of the source hospital** |  |
| **Not available** | **ID of the source hospital** |  |
|  | ***Following fields will get automatically updated with previously entered information*** | |
| **a4_Date_delivery** | Date of Delivery |  |
| **a5_place_delivery** | Place of Delivery (hospital/ home) |  |
| **deleted** | Name of hospital where delivered |  |
| **a7_periodofgestation** | Period of gestation when delivered |  |
| **a8_gender_baby** | Gender of the baby 1. Male 2. Female |  |
|  | ***Only for newborn recruited from Postnatal ward*** |  |
| **a10_complicationpncward_newborn** | Did the mother have any complication at the time of recruitment in postnatal ward? 1. Yes 2. No |  |
| **a9_complicationpncward_mother** | If yes, what was it? |  |
| **a10_complicationpncward_newborn** | Did the newborn have any complication at the time of recruitment in postnatal ward? 1. Yes 2. No |  |
| **a10_complicationpncward_newbor** | If yes, what was it? |  |
|  | ***Only for newborn recruited from SNCU ward*** |  |
| **a11_Admissiondatesncu** | Date of admission of the baby in the SNCU |  |
| **a12_age_admission** | Age (in days) at time of admission |  |
| **a13__diagnosis_admission1**  **a13__diagnosis_admission2 etc** | What was the diagnosis at admission? |  |
| **a14_Date_discharge_referral_lam** | Date of discharge / referral / LAMA if applicable |  |

**Make a call on the phone number provided. Try at least for three consecutive days i.e. day 7, day 8 & 9 for the call after 7 days and day 28, day 29, day 30 for call after 4 weeksbefore you label it not reachable.**

Greet the receiver of call and ask if you can talk to “Name of the mother”.

1. In case mother cannot be available then immediate care taker of the newborn shall be requested.
2. Once the mother whom you wish to talk is there on the other side, Confirm if it is the appropriate time to talk to her
3. Explain the project to her/him (use the information sheet)
4. Explain that the call is made to understand her experience about the care/services provided to mother and baby in the hospital
5. Ask for consent, read the consent form

|  | **Ask the respondent of the call** |  |
| --- | --- | --- |
| **A.15** | Name of respondent |  |
| **A.16** | Age of respondent |  |
| **A.17** | Relationship with the new born   1. Mother 2. Father 3. Grand parent 4. Other, specify |  |
| **A.18** | Consent obtained 1. Yes 2. No |  |

|  | ***Record these by asking mother/ care taker*** | **7 day** | 1. **ay** |
| --- | --- | --- | --- |
| **B.1**  b1_Date_discharge_mother | Date of discharge from PNC ward /SNCU ward |  | **b1_Date_discharge_28** |
| **B.2**  b2_mother_healthstatus | How is the mother doing?   1. Well 2. Sick 3. Dead   *In case of death of the woman, be sympathetic and record the following details* |  | **b2_mother_healthstatus_28 etc** |
| 1. **2.1**   b2_motherreason_death | What was the reason for the death of the mother?   1. Post-partum haemorrhage/Excessive bleeding 2. Eclampsia 3. Sepsis 4. Convulsions 5. Don’t Know 6. Others, please specify |  |  |
| **2.2**  b2_motherdate_death | Date of Death | DD/MM/YYYY |  |
| b3_baby_healthstatus | How is the baby doing?   1. Well 2. Sick 3. Dead   *In case of death of the baby, be sympathetic and record* |  |  |
| **3.1**  b3_babyreason_Sick_death | What was the reason for the death of the baby?   1. Prematurity 2. Inability/ Difficulty in Breathing 3. Meconium aspiration 4. Infection 5. Pneumonia 6. Congenital Malformation 7. Don’t Know 8. Others, please specify |  |  |
| **3.2**  b3_Babydate_death | Date of Death | DD/MM/YYYY |  |
| **In case of death of the mother or newborn console the respondents and stop here.** | | | |
|  |  | *After discharge from the hospital* | *In last 3 weeks* |
| **b4_mother_complication** | Did the mother face any complication? 1. Yes 2. No |  |  |
| **b4_excessivebleeding**  **b4_fever**  **b4_painabdomen**  **b4_fouldischarge**  **b4_convulsions**  **b4_swelling etc** | If yes, What was it? |  |  |
|  | 1. Excessive bleeding *(using more than 6 pads in a day after delivery)* |  |  |
|  | 1. Fever with Chills |  |  |
|  | 1. Pain in abdomen |  |  |
|  | 1. Foul smelling discharge |  |  |
|  | 1. Convulsions |  |  |
|  | 1. Swelling of face and hands |  |  |
|  | 1. Severe Headache |  |  |
|  | 1. Blurred vision |  |  |
|  | 1. Perineal Swelling |  |  |
|  | 1. Engorged/ Painful breast |  |  |
|  | 1. Sore/Cracked nipples |  |  |
|  | 1. Any other, please specify |  |  |
| **b4_advice_healthworker** | Did mother seek advice from any health worker regarding this complication?   1. Yes 2. No |  |  |
| **b4_type_healthworker** | If yes, From whom? |  |  |
|  | 1. ASHA |  |  |
|  | 1. ANM |  |  |
|  | 1. Doctor |  |  |
| **b5_asha_regularity** | Does ASHA visit you regularly?   1. Yes 2. No |  |  |
| **b5_asha_numbervisits** | When did she visit you last time? |  |  |
| **b5_ASHA_lasttime** | How many times has she visited after discharge? |  |  |
| **b6_newborn_complication** | Did the newborn have any complication?  1. Yes 2. No |  |  |
| **b6_newborn_illness** | If yes, what was the illness? |  |  |
|  | 1. Lethargy and poor sucking |  |  |
|  | 1. Inability/ Difficulty in Breathing |  |  |
|  | 1. Fever |  |  |
|  | 1. Pneumonia |  |  |
|  | 1. Hypothermia |  |  |
|  | 1. Failure to pass stool and urine |  |  |
|  | 1. Vomiting/diarrhea |  |  |
|  | 1. Yellow Palms & Soles |  |  |
| **b6_advice_healthworker** | If yes, did you seek advice from any health worker?  1. Yes 2. No |  |  |
| **b6_type_healthworker** | If yes, from whom? |  |  |
|  | 1. ASHA |  |  |
|  | 1. ANM |  |  |
|  | 1. Doctor |  |  |
| **b7_type_feed** | Is the baby on   1. Direct breast milk 2. Expressed breast milk, 3. Any other feed (skip to 11) |  |  |
| **b7_counsel_handwashing** | Were you counselled about washing your hands before feeding? 1. Yes 2. No |  |  |
| **b7_counsel_expressingmilk** | If on expressed milk, were you counselled about expressing the milk in the hospital? 1. Yes 2. No |  |  |
| **b7_counsel_cleancontainer** | Were you counselled about using a clean wide mouthed container for expressing milk? 1. Yes 2. No |  |  |
| **b8_anyotherfeedyn** | Is anything else also fed to the baby apart from breast milk? 1. Yes 2. No |  |  |
| **b9_anyotherfeed_specify** | If yes, Specify |  |  |
| **b10_counsel_positionbreastfeed**  **b10_counsel_exclusivebreastfee etc** | Were you counselled on any of the given topics at the time of discharge?  **(*1-Yes, 2-No, 3- Don’t Know*)** |  |  |
|  | Position of breastfeeding |  |  |
|  | Counselling on exclusive breast feeding for first six months |  |  |
|  | Advise for not bathing the child till the seventh day |  |  |
|  | Taking nutritious food in adequate amounts |  |  |
|  | Immunization advise for the newborn |  |  |
|  | Keeping the baby warm/Kangaroo mother care |  |  |
|  | Keeping the cord clean |  |  |
|  | Whom to contact in case of any newborn illness |  |  |
|  | Family Planning |  |  |
| **b11_counsel_excessivebleeding**  **b11_counsel_feverchills etc** | Were you counselled to identify the following complications at the time of discharge?  **(*1-Yes, 2-No, 3- Don’t Know*)** |  |  |
|  | ***That may develop in mother***   1. Excessive bleeding *(using more than 6 pads in a day after delivery)* |  |  |
|  | 1. Fever with Chills |  |  |
|  | 1. Pain in abdomen |  |  |
|  | 1. Foul smelling discharge |  |  |
|  | 1. Convulsions |  |  |
|  | 1. Swelling of face and hands |  |  |
|  | 1. Severe Headache |  |  |
|  | 1. Blurred vision |  |  |
|  | 1. Perineal Swelling |  |  |
|  | 1. Engorged/ Painful breast |  |  |
|  | 1. Sore/Cracked nipples |  |  |
|  | 1. Any other, please specify |  |  |
|  | ***That may develop in baby*** |  |  |
|  | 1. Lethargy and poor sucking |  |  |
|  | 1. Respiratory problems |  |  |
|  | 1. High temperature |  |  |
|  | 1. Hypothermia |  |  |
|  | 1. Failure to pass meconium and urine |  |  |
|  | 1. Vomiting/diarrhea |  |  |
|  | 1. Yellow Palms & Soles |  |  |
| b12_paydelivery  b12_paydrugs etc | Did you have to pay at hospital for any of the following service?  ***Multiple responses possible*** |  |  |
|  | 1. Delivery |  |  |
|  | 1. Drugs |  |  |
|  | 1. Diagnostics |  |  |
|  | 1. Transport |  |  |
|  | 1. Diet |  |  |
|  | 1. Other, please specify |  |  |
| ***Only for the new-borns recruited from SNCU*** | | | |
|  |  | *After discharge from the hospital* | *In last 3 weeks* |
| b13_sncufollowup | Is there any follow-up from the SNCU? |  |  |
| b14_revisitsncu | Did you have to revisit the SNCU for the same ailment? 1. Yes 2. No |  |  |
| ***Note any other information shared by the mother*** | | | |
